# Dominant negative variants in *ITPR3* impair T cell Ca^2+^ dynamics causing combined immunodeficiency

**DOI:** 10.1084/jem.20220979

**Published:** 2024-11-19

**Authors:** Elena Blanco, Carme Camps, Sameer Bahal, Mohit D. Kerai, Matteo P. Ferla, Adam M. Rochussen, Adam E. Handel, Zainab M. Golwala, Helena Spiridou Goncalves, Susanne Kricke, Fabian Klein, Fang Zhang, Federica Zinghirino, Grace Evans, Thomas M. Keane, Sabrina Lizot, Maaike A.A. Kusters, Mildred A. Iro, Sanjay V. Patel, Emma C. Morris, Siobhan O. Burns, Ruth Radcliffe, Pradeep Vasudevan, Arthur Price, Olivia Gillham, Gabriel E. Valdebenito, Grant S. Stewart, Austen Worth, Stuart P. Adams, Michael Duchen, Isabelle André, David J. Adams, Giorgia Santili, Kimberly C. Gilmour, Georg A. Holländer, E. Graham Davies, Jenny C. Taylor, Gillian M. Griffiths, Adrian J. Thrasher, Fatima Dhalla, Alexandra Y. Kreins

**Affiliations:** 1 Molecular and Cellular Immunology, Great Ormond Street Institute of Child Health, University College London, London, UK; 2 National Institute for Health Research Oxford Biomedical Research Centre, University of Oxford, Oxford, UK; 3 Wellcome Centre for Human Genetics, University of Oxford, Oxford, UK; 4 Immunology Laboratory, Great Ormond Street Hospital for Children NHS Foundation Trust, London, UK; 5 Cambridge Institute for Medical Research, University of Cambridge, Cambridge, UK; 6 Department of Paediatrics and Institute of Developmental and Regenerative Medicine, University of Oxford, Oxford, UK; 7 Department of Paediatric Immunology and Gene Therapy, Great Ormond Street Hospital for Children NHS Foundation Trust, London, UK; 8 SIHMDS-Haematology Laboratory, Great Ormond Street Hospital for Children NHS Foundation Trust, London, UK; 9 Wellcome Sanger Institute, Cambridge, UK; 10 European Molecular Biology Laboratory, European Bioinformatics Institute, Wellcome Genome Campus, Cambridge, UK; 11 Human Lymphohematopoiesis Laboratory, Imagine Institute, INSERM UMR 1163, Université Paris Cité, Paris, France; 12 Department of Paediatric Infectious Diseases and Immunology, University Hospital Southampton NHS Foundation Trust, Southampton, UK; 13 Faculty of Medicine and Institute of Life Sciences, University of Southampton, Southampton, UK; 14 Department of Immunology, Royal Free London Hospitals NHS Foundation Trust, London, UK; 15 Institute for Immunity and Transplantation, University College London, London, UK; 16 Department of Immunology, University Hospitals of Leicester NHS Trust, Leicester, UK; 17 Department of Clinical Genetics, University Hospitals of Leicester NHS Trust, Leicester, UK; 18 Department of Cell and Developmental Biology and Consortium for Mitochondrial Research, University College London, London, UK; 19 Institute of Cancer and Genomic Sciences, University of Birmingham, Birmingham, UK; 20 Paediatric Immunology, Department of Biomedicine, University of Basel and University Children’s Hospital, Basel, Switzerland; 21 Department of Biosystems Science and Engineering, ETH Zurich, Basel, Switzerland; 22 Department of Clinical Immunology, Oxford University Hospitals NHS Foundation Trust, Oxford, UK; 23 Institute for Health Research Great Ormond Street Hospital Biomedical Research Centre, London, UK

## Abstract

The importance of calcium (Ca^2+^) as a second messenger in T cell signaling is exemplified by genetic deficiencies of *STIM1* and *ORAI1*, which abolish store-operated Ca^2+^ entry (SOCE) resulting in combined immunodeficiency (CID). We report five unrelated patients with de novo missense variants in *ITPR3*, encoding a subunit of the inositol 1,4,5-trisphosphate receptor (IP_3_R), which forms a Ca^2+^ channel in the endoplasmic reticulum (ER) membrane responsible for the release of ER Ca^2+^ required to trigger SOCE, and for Ca^2+^ transfer to other organelles. The patients presented with CID, abnormal T cell Ca^2+^ homeostasis, incompletely penetrant ectodermal dysplasia, and multisystem disease. Their predominant T cell immunodeficiency is characterized by significant T cell lymphopenia, defects in late stages of thymic T cell development, and impaired function of peripheral T cells, including inadequate NF-κB- and NFAT-mediated, proliferative, and metabolic responses to activation. Pathogenicity is not due to haploinsufficiency, rather ITPR3 protein variants interfere with IP_3_R channel function leading to depletion of ER Ca^2+^ stores and blunted SOCE in T cells.

## Teaser

Dominant negative variants in *ITPR3*, encoding a subunit of the IP_3_ receptor, cause Ca^2+^ channel leakiness resulting in ER store depletion and impaired T cell Ca^2+^ dynamics. Patients present with incompletely penetrant syndromic combined immunodeficiency, ectodermal dysplasia, and multisystem disease.

## Introduction

Calcium (Ca^2+^) signaling is a universal second messenger system that is highly conserved in evolution. Amongst its pleiotropic roles, Ca^2+^ signaling in lymphocytes is essential for T cell activation and function including cell motility, immune synapse formation, cytotoxic granule release, cytokine and chemokine production, proliferation, differentiation, metabolism, and apoptosis (Feske, 2007; Feske et al., 2012; Trebak and Kinet, 2019; Vaeth et al., 2020). Signaling via the T cell receptor (TCR) and G-protein coupled receptors leads to the activation of phospholipase C isozymes, which hydrolyze membrane phosphatidylinositol-4,5-bisphosphate (PIP_2_) to generate inositol 1,4,5-trisphosphate (IP_3_) and diacylglycerol (Newton et al., 2016). The receptor for IP_3_ (IP_3_R) is assembled from homo- or heterotetramers of three different ITPR homologs (ITPR1, ITPR2, and ITPR3), forming a ubiquitously expressed Ca^2+^ channel (Ouyang et al., 2014). IP_3_R is located in the membrane of the endoplasmic reticulum (ER), the major intracellular site of Ca^2+^ storage. On binding of IP_3_, the IP_3_R channel opens to allow Ca^2+^ stores to be rapidly unloaded along a steep concentration gradient from the ER into the cytoplasm through a mechanism called internal storage release (Boehning and Joseph, 2000; Fan et al., 2015; Seo et al., 2012; Yamazaki et al., 2010). Depletion of ER Ca^2+^ stores is sensed by ER-resident STIM proteins, which upon conformational changes are able to bind to and open Ca^2+^ release–activated channels (CRAC) formed by ORAI proteins within the plasma membrane (Feske, 2007; Feske et al., 2006; Liou et al., 2005; Prakriya et al., 2006; Soboloff et al., 2006). The result is Ca^2+^ influx from the extracellular space into the cytosol, again driven by a steep concentration gradient, in a process termed store-operated Ca^2+^ entry (SOCE) due to its dependence on the prior depletion of ER Ca^2+^ stores (Zweifach and Lewis, 1993). Elevated concentrations of cytosolic Ca^2+^ subsequently serve to activate Ca^2+^-dependent enzymes and transcription factors, including NFAT and NF-κB, and influence mitochondrial functions (Feske, 2007; Feske et al., 2012; Trebak and Kinet, 2019; Vaeth et al., 2020). Aside from their role in triggering SOCE, IP_3_Rs also function to transfer Ca^2+^ to other intracellular organelles at sites of physical contact with the ER, termed “membrane contact sites” (Ahumada-Castro et al., 2021). IP_3_R-mediated Ca^2+^ transfer from the ER to endosomes, lysosomes, and mitochondria regulates their functions, alters various pathways of cellular metabolism, and influences cell survival, autophagy, and apoptosis (Ahumada-Castro et al., 2021; Decuypere et al., 2011; Rieusset, 2018).

The critical importance of Ca^2+^ signaling, in particular SOCE, in immune system function and regulation is exemplified by inborn errors of immunity (IEI), designated “CRAC channelopathies,” due to autosomal recessive loss-of-function (LOF) variants in *STIM1* and *ORAI1*, causing abolished SOCE (Badran et al., 2016; Byun et al., 2010; Feske et al., 2006, 2010; Fuchs et al., 2012; Lacruz and Feske, 2015; Lian et al., 2018; McCarl et al., 2009; Picard et al., 2009; Schaballie et al., 2015; Vaeth et al., 2017; Yu et al., 2021). Patients present with early-onset life-threatening combined immune deficiency (CID) due to defective lymphocyte function rather than impaired T cell development. Circulating T-lymphocytes are typically normal or elevated in number in ORAI1- and STIM1-deficient patients, but are impaired in their activation, proliferation, cytokine production, and metabolism. The majority of these patients suffer from recurrent infections, in particular severe viral infections, often requiring corrective treatment with hematopoietic cell transplantation (HCT). Other clinical features include autoimmunity, muscular hypotonia, osteopetrosis, and anhidrotic ectodermal dysplasia (ED) with prominent dental enamel defects (Badran et al., 2016; Byun et al., 2010; Feske et al., 2006, 2010; Fuchs et al., 2012; Lacruz and Feske, 2015; Lian et al., 2018; McCarl et al., 2009; Picard et al., 2009; Schaballie et al., 2015; Vaeth et al., 2017; Yu et al., 2021). Recently, compound heterozygous variants in *ITPR3* have been reported in two unrelated patients with CID of variable severity (Neumann et al., 2023). One patient had early onset severe CID requiring HCT during childhood, whilst the second presented as a young adult with a milder clinical and immunological phenotype. In the single untransplanted patient, partial defects in T cell Ca^2+^ signaling, NFAT translocation, ERK phosphorylation, and proliferation were documented (Neumann et al., 2023).

Here, we report five unrelated patients with monoallelic variants in *ITPR3* and clinical and immunological features that overlap with CRAC channelopathies. We comprehensively profile their immunophenotype and provide functional data in primary T cells, patient-derived cytotoxic T-lymphoblasts (CTLs), and gene-edited T cell lines showing impaired Ca^2+^ signaling and defects in activation, proliferation, metabolic reprogramming, and T cell development.

## Results

### Patient clinical characteristics

**Figure 1.**
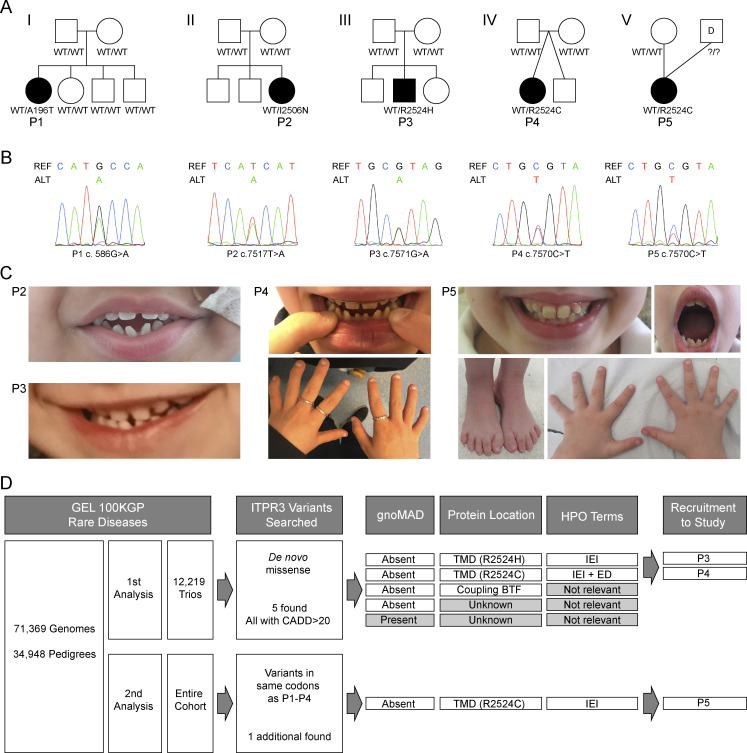
**Identification of de novo *ITPR3* variants in five unrelated patients presenting with CID and ED. (A)** Pedigrees of the five unrelated patients included in this study. Females are represented by circles, males by squares, and affected individuals (P1–P5) are denoted by filled, black symbols. P4 (IV) is part of a dizygotic twinship. P5 (V) was conceived using donor sperm. Where known, genotypes are displayed below each individual. **(B)** Sanger sequencing of genomic DNA confirming the heterozygous *ITPR3* variants found in P1–P5; REF = reference sequence, ALT = alternative sequence; representative of two independent experiments. **(C)** Clinical photographs of P2, P3, P4, and P5 demonstrate some of the associated syndromic features, including dental abnormalities (microdontia, hypodontia, conical teeth, and enamel hypoplasia), nail dystrophy, and deformity of the feet and hands. **(D)** Flowchart detailing the strategy used to identify patients with heterozygous *ITPR3* variants within the rare diseases cohort of the GEL 100,000 Genomes Project (GEL100KGP); Trios = genomes of probands and parents.

We identified four heterozygous *ITPR3* variants in five patients (P1–P5) from five unrelated kindreds in the United Kingdom (Fig. 1, A and B). Their clinical and immunological characteristics are summarized in Table 1 and Table 2, respectively (also see Case reports in Materials and methods). In brief, all five patients were born to non-consanguineous parents and presented during childhood, most often with recurrent viral and bacterial respiratory infections, but also non-respiratory viral infections, mainly due to DNA viruses. Three patients had features of immune dysregulation, including immune thrombocytopenia (ITP), lymphoproliferation, and atopy. At initial immunological assessment, all were lymphopenic with extremely low T cell counts and moderate B cell lymphopenia associated with mild hypogammaglobulinemia but preserved responses to vaccination. Counts of naïve T cells and TCR-excision circles (TRECs) were low, TCR V beta (TCRVβ) repertoires were restricted, and proliferation in response to phytohaemagglutinin (PHA) was impaired. The lymphopenia persisted over time, but the clinical severity of the immunodeficiency has been variable among patients. Two patients underwent HCT for CID at the ages of 9 and 3 years, and two additional patients developed EBV-driven lymphomas at the ages of 22 and 12 years, requiring chemotherapy followed by HCT. The fifth patient is 18 years old and clinically well on conservative management with antibiotic prophylaxis. In addition to their immunodeficiency, variable degrees of ED were noted in all but one patient (Fig. 1 C), including micro- and hypodontia with conical incisors, thin hair and nails, and hypohidrosis. In the patients with more severe ED, impaired NF-*κ*B signaling was suspected but cytokine production in response to Toll-like receptor (TLR) agonists was not affected (data not shown). Additionally, three out of the five patients suffer from considerable neurological, gastrointestinal, and/or respiratory complications. Genetic investigations were initially inconclusive, and these five patients remained with a diagnosis of genetically undefined CID, variably associated with ED and/or multisystem disease.

**Table 1.** Genetic and clinical characteristics of patients with heterozygous *ITPR3* mutations

PATIENT	P1	P2		P3	P4	P5
Variant annotation					
Inheritance	het, de novo	het, de novo	het, maternal	het, de novo	het, de novo	het, ? de novo
Nt change	c.586G>A	c.7517T>A	c.5549G>A	c.7571G>A	c.7570C>T	c.7570C>T
AA change	p.A196T	p.I2506N	p.R1850Q	p.R2524H	p.R2524C	p.R2524C
GNOMAD, DBSNP151, 1000G	No	No	gnomAD freq = 0.082 6079 hom	No	No	No
CADD (95% CI MSC = 16)	32	33	21.5	32	30	30
SIFT	Deleterious	Deleterious	Tolerated	Deleterious	Deleterious	Deleterious
Polyphen2	Probably damaging	Probably damaging	Benign	Probably damaging	Probably damaging	Probably damaging
Clinical presentation					
Age at presentation	7 yo	3 mo		Infancy	4 mo	14 mo
Age now	24 yo	6 yo		33 yo	18 yo	13 yo
Combined immunodeficiency	Yes	Yes		Yes	Yes	Yes
Viral infections	Cutaneous (severe HPV, resistant molluscum); EBV	Respiratory (RSV); GI (adenovirus)		Respiratory; cutaneous (severe VZV); EBV	Respiratory (VZV, adenovirus, RSV); cutaneous (HPV)	Respiratory; EBV
Bacterial infections	Impetigo	LRTI (*H. influenzae*)		LRTI	*P. aeruginosa* resp. colonization	LRTI (*H. influenzae*, *S. pneumoniae*)
T cell lymphopenia	Yes	Yes		Yes	Yes	Yes
B cell lymphopenia	No	No		Yes	Yes	Yes
Hypogammaglobulinemia	No	Yes		Yes	No	No
Other	ITP, eczema			Atopy, lymphoma		ITP, lymphoma
ED	No	Yes		Yes	Yes	Yes
Dental anomalies	No	Small, peg-shaped lower incisors		Hypo- and microdontia	Peg-shaped lower incisors, hypodontia	Peg-shaped lower incisors, enamel discoloration
Hair anomalies	No	Thin hair		Thin hair	Thin, light hair	Thin, light hair
Nail anomalies	No	No		No	Thin nails	Slow nail growth
Hypohidrosis	No	No		No	Yes	No
Other system disease	No	Yes		No	Yes	Yes
Neurology		Unsafe swallow, chronic motor neuronopathy				Demyelinating motor and sensory neuropathy
Gastroenterology		Chronic constipation				
Respiratory					Bronchiectasis	Bronchiectasis
Other					Atrial septal defect	Growth restriction, developmental delay, dysmorphism
Current treatment	Post-HCT, Abx, IgRT	Post-HCT, CSA, Abx, IgRT		Post-HCT	Abx, DNAse nebulizers	Post-HCT, Abx, IgRT

95% CI MSC, 95% confidence interval MSC; Abx, antibiotics; CSA, cyclosporin; GI, gastrointestinal; het, heterozygous; hom, homozygous; HPV, human papilloma virus; IgRT, immunoglobulin replacement therapy; LRTI, lower respiratory tract infection; mo, months old; Nt, nucleotide; Resp, respiratory; RSV, respiratory syncytial virus; VZV, varicella zoster virus; yo, years old.

**Table 2.** Immunological assessment

Initial assessment	P1 (7 yo)	P2 (6 mo)	P3 (11 yo)	P4 (2 yo)	P5 (6 yo)
FBC					
Lymphocytes/µl	**780** (1,500–7,000)	**1,080** (3,000–13500)	**530** (1,200–5,200)	1,600 (6,000–9,000)	**800** (1,500–6,500)
Neutrophils/µl	7,510 (1,500–8,000)	3,280 (1,000–8,500)	2,480 (1,800–8,000)	1,900 (1,500–8,000)	**9,860** (1,500–8,000)
Eosinophils/µl	330 (100–800)	160 (100–300)	310 (100–800)	600 (200–1,000)	90 (40–400)
Platelets/µl	**109k** (150k–450k)	318k (150k–450k)	**142k** (150k–450k)	327k (150k–400k)	**478k** (140k–400k)
LSS					
CD3^+^ cells/µl	**170** (1,352–3,275)	**230** (3,764–6,289)	**430** (930–3,477)	**530** (852–5,333)	**580** (1,200–2,600)
CD4^+^ cells/µl	**10** (776–1,815)	**130** (2,093–4,769)	**290** (576–1,891)	**360** (516–3,448)	**270** (650–1,500)
CD8^+^ cells/µl	**120** (366–1,171)	**100** (720–1,271)	**130** (261–1,189)	**100** (188–1,805)	**300** (370–1,100)
CD4^+^CD45RA^+^CD27^+^ cells/µl	**0** (424–1,393)	**10** (1,748–4,201)	**90** (264–1,484)	NA	NA
CD8^+^CD45RA^+^CD27^+^ cells/µl	**86** (175–730)	**10** (564–1,040)	**85** (94–986)	NA	NA
CD16^+^CD56^+^ cells/µl	280 (106–1,348)	530 (237–1,146)	**20** (109–1,021)	590 (138–1,759)	410 (100–480)
CD19^+^ cells/µl	**260** (157–725)	**280** (896–2,316)	**40** (173–1,194)	680 (232–1,637)	**240** (270–860)
TRECS/10^6^ T cells	**Absent**	**1,028** (10th %: 15,337, median: 35,982)	**7,797** (10th %: 8,538, median: 22,214)	NA	NA
Spectratyping	**Abnormal**	**Abnormal**	**Abnormal**	NA	NA
PHA response	**Absent**	**Absent**	**Impaired**	Borderline^a^	**Impaired**
IgG (g/liter)	12.50 (5.4–16.1)	**2.75** (3–9)	**4.64** (6–16)	15.2 (6–16)	9.0 (4.9–16.1)
IgA (g/liter)	1.29 (0.7–2.5)	0.19 (0.15–0.7)	2.57 (0.8–2.8)	1.8 (0.8–2.8)	3.68 (0.50–2.4)
IgM (g/liter)	0.63 (0.5–1.8)	**0.08** (0.4–1.6)	**0.47** (0.5–1.9)	**0.3** (0.5–1.9)	**0.37** (0.5–1.8)
Vaccine responses^b^	Normal	NA	Normal	Normal	Normal
POST-HCT	**P1 (14 ** **years ** **post; 24 yo)**	**P2 (2 y** **ears** ** post; 6 yo)**	**P3 (10 ** **years** ** post; 32 yo)**		**P5 (6 mo post; 13 yo)**
T cell donor chimerism	41%	100%	91%		100%
Lymphocytes/µl	1,001 (895–3,684)	2,110 (1,827–4,564)	1,102 (895–3,684)		**930** (1,238–4,792)
LSS					
CD3^+^ cells/µl	780 (564–2,935)	1,640 (1,352–3,275)	735 (564–2,935)		**690** (930–3,477)
CD4^+^ cells/µl	390 (207–1,900)	970 (776–1,815)	396 (207–1,900)		**510** (576–1,891)
CD8^+^ cells/µl	320 (160–1,103)	**320** (366–1,171)	339 (160–1,103)		**160** (261–1,189)
TRECS/10^6^ T cells	4,225 (10th %: NA; median: 4,120)	9,710 (10th %: 8,538, median: 22,214)	4,418^c^ (10th %: NA, median: 4,120)		**2,080** (10th %: NA, median: 15,284)
Spectratyping	Normal	Normal	**Abnormal** ^c^		**Improved**
PHA response	Normal	Normal	Normal		Normal

Laboratory age-matched reference ranges given in parentheses. Results that fall outside of their respective reference ranges are marked in bold. 10th %, 10th centile; FBC, full blood count; k, thousand; LSS; lymphocyte subsets; mo, months old; NA, not available; PHA, phytohemagglutinin; TRECs; TCR excision circles; yo, years old.

a P4 proliferative response of 12,972 counts per minute (cpm) versus travel control 28,147 cpm (laboratory normal cut off at 12,500 cpm).

b Assessed prior to the commencement of immunoglobulin replacement therapy.

c Performed at 7 years post-HCT.

### Identification of de novo *ITPR3* variants in five patients with CID

We initially investigated two patients, P1 and P2, who presented at 7 years and 6 mo of age, respectively, with significant CID (Table 1). P2 additionally had features of ED (Fig. 1 C) and multisystem disease (Table 1). P1 was born to non-consanguineous parents and had three unaffected siblings. In the absence of a genetic diagnosis after sequencing a panel of known IEI genes, whole exome sequencing (WES) was performed on the sextet. The only disease-segregating private variant identified in P1 was a de novo missense variant in *ITPR3* (NC_000006.12:g.33659078G>A; NM_002224.3:c.586G>A; NP_002215.2:p.A196T), the gene encoding the type 3 member of the receptor for IP_3_ (IP_3_R3) (Fig. 1, A and B). P2 was born to non-consanguineous parents and has two unaffected siblings. Trio-based whole genome sequencing (WGS) performed after standard diagnostic approaches, including microarray comparative genomic hybridization, IEI gene panel, and clinical WES, did not yield a genetic diagnosis. Variants were filtered according to quality, rarity, non-synonymous effect, and mode of inheritance. Coding variants were prioritized according to Combined Annotation Dependent Depletion (CADD) score and GeneNetwork Assisted Diagnostic Optimization (GADO) score computed from Human Phenotype Ontology (HPO) terms describing the phenotype of the patient (Deelen et al., 2019; Kircher et al., 2014). Using this approach, a de novo missense variant in *ITPR3* (NC_000006.12:g.33692786T>A; NM_002224.3:c.7517T>A; NP_002215.2:p.I2506N) was identified as the top ranking candidate (Fig. 1, A and B). The de novo *ITPR3* variants identified in P1 and P2 have not been reported in any public databases (gnomAD v4.1, dbSNP151, 1000G) and are predicted to be deleterious or probably damaging by SIFT and Polyphen2, respectively (Table 1). A polymorphism in *ITPR3* was additionally found in P2 (NC_000006.12:g.33685709G>A; NM_002224.3: c.5549G>A; NP_002215.2:p.R1850Q). This variant was inherited from her unaffected mother and has an allele frequency of 0.082 in gnomAD, including 6,079 homozygotes (Table 1). It was not possible to phase this polymorphism in relation to the de novo I2506N variant due to the short read length of the sequencing technology used.

The important role of Ca^2+^-mediated signaling in lymphocyte biology (Feske, 2007; Feske et al., 2012) and the strong phenotypic overlap, in particular between P2 and STIM1- and ORAI1-deficient patients and two recently reported patients with compound heterozygous *ITPR3* variants, consolidated *ITPR3* as a promising candidate gene (Badran et al., 2016; Byun et al., 2010; Feske et al., 2006, 2010; Fuchs et al., 2012; Lian et al., 2018; McCarl et al., 2009; Neumann et al., 2023; Picard et al., 2009; Schaballie et al., 2015; Yu et al., 2021). We therefore next investigated whether variants in *ITPR3* could be found in more cases of genetically undefined IEIs by interrogating the Genomics England (GEL) 100,000 Genomes Project Rare Diseases cohort which comprises the genomes from 71,369 participants (see Materials and methods) (Caulfield et al., 2017). Since the candidate variants identified in P1 and P2 were de novo, we first searched for de novo missense variants in *ITPR3* in the probands of 12,219 trios available within the GEL Rare Diseases cohort (Fig. 1 D). We found five such variants, all with CADD scores above 20. Three variants were however discarded, with one being reported in gnomAD with a frequency of 0.00059 and all three occurring in probands for whom the HPO terms did not indicate a possible IEI or ED. The submitted HPO terms for the remaining two patients suggested IEI^+/−^, ED, in common with P1 and P2. Hence, our search resulted in the identification of two patients of interest, P3 and P4, with de novo *ITPR3* variants affecting the same amino acid (AA) residue at position 2524 (respectively NC_000006.12:g. 33692840G>A; NM_002224.3:c.7571G>A; NP_002215.2:p.R2524H in P3 and NC_000006.12:g. 33692839C>T; NM_002224.3:c.7570C>T; NP_002215.2:p.R2524C in P4) that are absent in population databases (gnomAD v4.1, dbSNP151, 1000G) and are predicted to be deleterious by SIFT and probably damaging by Polyphen2 (Table 1; and Fig. 1, A and B). The R2524C variant has been reported in two patients with early onset Charcot-Marie-Tooth (CMT) disease, including one patient with early onset CID and ED who additionally inherited the R1850Q polymorphism in *trans* from a clinically unaffected parent (Neumann et al., 2023; Rönkkö et al., 2020; Terry et al., 2022). We subsequently screened the entire GEL cohort for the presence of variants affecting the same codons as those altered in P1–P4 (i.e., encoding AA196, AA2506, and AA2524) (Fig. 1 D). As a result, an additional patient, P5, carrying the same *ITPR3* variant as P4 was identified (Fig. 1, A and B). HPO terms again suggested IEI. Paternal DNA was not available for sequencing and the variant was not found in the maternal sample. Although ED had not been included in the submitted HPO terms for P3 and P5, upon further review, P3, P4, and P5 had all been diagnosed clinically with CID and ED (Fig. 1 C). No other genetic variants were identified that would provide a molecular explanation for their clinical presentation other than the *ITPR3* variants (Caulfield et al., 2017).

In summary, we identified four de novo missense variants in *ITPR3* in five unrelated patients with childhood-onset CID and incomplete penetrance for ED and multisystem disease through WES and WGS with confirmation by Sanger sequencing (Fig. 1 B). All variants have high CADD scores (≥30), well above the mutation significance cutoff (MSC) score for *ITPR3* of 16 (lower limit of 95% CI) (Itan et al., 2016), and are absent from public databases (Table 1). In P2, but not in the four other patients, a polymorphism was also found, which was maternally inherited.

### ITPR3 is expressed in patient cells

**Figure 2.**
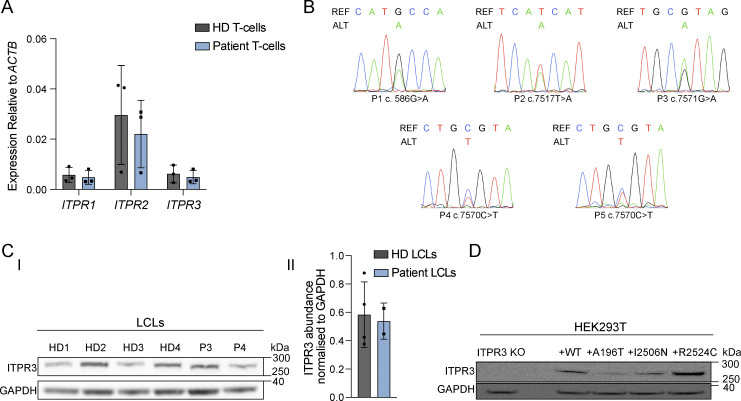
**Expression of ITPR members in HD and patient cells. (A)** RT-qPCR for *ITPR1*, *ITPR2*, and *ITPR3* transcripts in peripheral blood T cells isolated from HD and patients (P2, P4, and P5). Results shown are relative to beta-actin (*ACTB*) expression, are representative of two independent experiments, and are not statistically significant via multiple unpaired *t* tests with Holm-Sidak correction for multiple comparisons. **(B)** Sanger sequencing of cDNA showing expression of WT and variant *ITPR3* transcripts for P1 (fibroblasts), P2 (T cells), P3 (LCLs), P4 (T cells), and P5 (T cells); REF = reference sequence, ALT = alternative sequence; representative of two independent experiments. **(C)** Protein expression of ITPR3 in LCLs from HDs, P3 and P4. **(I)** Western blot for ITPR3 and GAPDH using LCL lysates; representative of three independent experiments. **(II)** Densitometric quantification of ITPR3 protein abundance in LCLs relative to GAPDH. **(D)** Western blot for ITPR3 and GAPDH in ITPR3 KO HEK293T cells (ITPR3 KO) and in transfected ITPR3 KO cells expressing WT ITPR3 protein or the A196T, I2506N, and R2524C protein variants; representative of two independent transfection experiments. Source data are available for this figure: SourceData F2.

**Figure S1.**
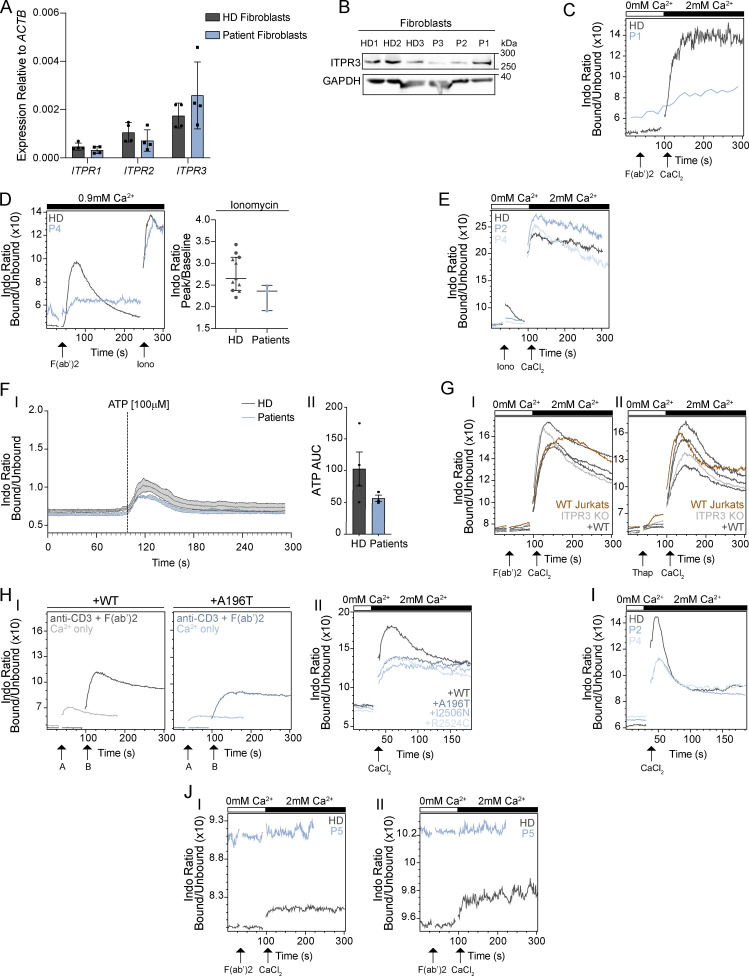
**Ca**^**2+**^ **flux in patient primary skin fibroblasts, primary T** **cells, and gene-edited cell lines expressing patient protein variants. (A)** RT-qPCR for *ITPR1*, *ITPR2*, and *ITPR3* transcripts in primary skin fibroblasts obtained from three HDs and three patients (P1, P2, and P3). Results shown are relative to beta-actin (*ACTB*) expression are representative of two independent experiments and are not statistically significant via multiple unpaired *t* tests with Holm–Sidak correction for multiple comparisons. **(B)** Western blot for ITPR3 and GAPDH in primary skin fibroblasts from three HDs and three patients (P1, P2, and P3); representative of two independent experiments. **(C–J)** The ratiometric Ca^2+^ indicator Indo-1 was used to measure cytoplasmic Ca^2+^ concentration. **(C)** Ca^2+^ flux after stimulation with anti-CD3 plus F(ab′)2 in thawed pre-HCT primary T cells from P1 (blue) and from a HD (grey) in Ca^2+^-free media and upon addition of CaCl_2_. This single, independent experiment was analyzed separately from the data shown in Fig. 4 A due to poor cell viability. **(D)** Ca^2+^ flux after stimulation with anti-CD3/F(ab′)2 in primary T cells from P4 (blue) and from a HD (grey) in Ca^2+^-containing media and upon addition of ionomycin at 240 s; representative of three independent experiments; not significant, Mann–Whitney U test. **(E)** Ca^2+^ flux after stimulation with ionomycin in Ca^2+^-free media and upon addition of CaCl_2_ in CTLs from P2 and P4 (light blue) versus one HD; representative of two independent experiments. **(F)** Ca^2+^ flux in fibroblasts from four HDs (grey) versus P1, P2, and P3 (blue) stimulated with ATP; traces are shown in I and graph of the AUC of the peaks in II. Graphs represent the mean of three separate experiments and error bars indicate SEM. **(G)** Ca^2+^ flux in WT (red) and ITPR3 KO Jurkat T cells (light grey), and ITPR3 KO cells stably transduced with WT ITPR3 (dark grey) after stimulation with (I) anti-CD3/F(ab′)2 or (II) thapsigargin in Ca^2+^-free media and upon addition of CaCl_2_; representative of two independent experiments. **(H)** Ca^2+^ flux upon addition of CaCl_2_ in Ca^2+^-free media in (H) Jurkat ITPR3KO T cells expressing WT or variant ITPR3 proteins; (I) shows representative plots of ITPR3 WT (left) and variant expressing (right) cells either pre-stimulated with anti-CD3 and addition of F(ab′)2 (at timepoint A) followed by addition of CaCl_2_ (at timepoint B; darker line), or CaCl_2_ alone (at timepoint A; paler line); (II) shows a comparison of Ca^2+^ influx upon addition of CaCl_2_ alone (i.e., without agonist stimulation) in cells expressing WT ITPR3 or the various ITPR3 variant proteins; representative of two independent experiments. **(I)** Ca^2+^ flux upon addition of CaCl_2_ in Ca^2+^-free media in CTLs from P2, P4, and one HD; representative of two independent experiments. **(J)** Ca^2+^ flux in ex vivo differentiated T cells from P5 and from one HD stimulated with anti-CD3/F(ab′)2 in (I) single positive CD4^+^ and (II) single positive CD8^+^ cells; representative of two independent experiments. Source data are available for this figure: SourceData FS1.

The three ITPR proteins share ∼75% AA sequence identity (Gambardella et al., 2020; Prole and Taylor, 2019; Taylor et al., 1999). Although they are ubiquitously expressed, differences in tissue distribution, subcellular localization, and biophysical properties have been noted (Gambardella et al., 2020; Mangla et al., 2020; Prole and Taylor, 2019). All three proteins are expressed in lymphoid cells and functional redundancy has been demonstrated between them, at least in the context of murine thymocyte development (Gambardella et al., 2020; Monaco et al., 2019; Ouyang et al., 2014; Schmiedel et al., 2018). We found all three *ITPR*s to be transcribed in peripheral T cells and, at a lower level, in skin fibroblasts isolated from healthy donors (HD) (Fig. 2 A and Fig. S1 A). Notably, *ITPR3* RNA was detected in primary T cells and fibroblasts from patients at levels comparable to that of HD (Fig. 2 A and Fig. 1 A), with both WT and variant *ITPR3* transcripts being present (Fig. 2 B). Due to profound lymphopenia, it was not possible to assess ITPR3 protein expression in primary lymphocytes. We thus generated patient-derived lymphoblastoid cell lines (LCLs) from blood following EBV transformation. In patient LCLs, we found ITPR3 protein expression levels comparable with that of control LCLs (Fig. 2 C). We similarly found normal ITPR3 expression in patient skin fibroblasts (Fig. S1 B). To investigate the impact of the patient variants on protein expression, we knocked out *ITPR3* by CRISPR-Cas9 in human embryonic kidney 293T (HEK293T) cells and transiently transfected these knockout (KO) HEK293T cells with a plasmid carrying the WT *ITPR3* cDNA, or cDNA corresponding to p.A196T (P1), p.I2506N (P2), and p.R2524C (P4–P5; P3 being affected in the same AA). Western blotting detected variant protein products with the same apparent molecular weights as WT ITPR3 (Fig. 2 D).

Taken together, these results confirm that the missense variants in *ITPR3* present in the patients are transcribed, are not subject to premature RNA decay, lead to stable expression of protein variants, and do not significantly alter overall levels of ITPR3 protein expression.

### ITPR3 protein variants are predicted to impair IP_3_R channel function

**Figure 3.**
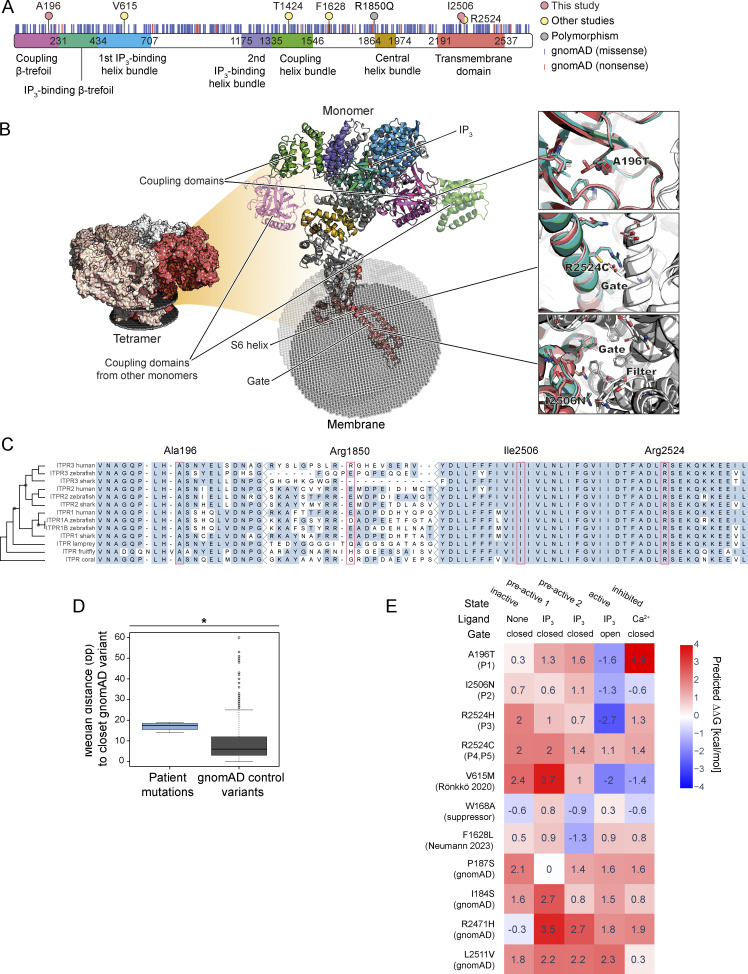
**In silico modeling of *ITPR3* variants. (A)** ITPR3 secondary structure with domains annotated. The location of the AAs affected by dominant variants are marked by pink circles (this study i.e., A196 in P1, I2506 in P2, and R2524 in P3–P5); and yellow circles (other studies [Neumann et al., 2023; Rönkkö et al., 2020; Terry et al., 2022]: V615, T1424, F1628, and R2524); the R1850Q polymorphism is marked by a grey circle. Missense and nonsense variants in gnomAD database are indicated by blue and red vertical lines, respectively. **(B)** ITPR3 tertiary structure with domains colored as indicated in panel A. The center shows the monomer of the cryo-EM structure 6DRC colored by domains as predicted by Pfam, with minor corrections in the ranges, namely coupling BTF (AA 3–230), IP_3_ binding BTF (AA 233–433), first IP_3_ binding helix bundle (437–707), second IP_3_ binding helix bundle (1175–1334), coupling helix bundle (1335–1546), central helix bundle (1864–1974), and ion channel transmembrane domain (2191–2537). The latter contains, within its S6 helix, a cationic selectivity filter (formed by N2472 and D2478), which favors the passage of Ca^2+^ over other cations, and a gate (formed by I2517 and F2513) to prevent ions from passing unless the channel is activated (Paknejad and Hite, 2018). The three insets (right) show the overlay of the WT (turquoise) and the variant (salmon) chains with other chains shown in white. **(C)** Multiple sequence alignment of AA sequences in orthologous ITPR proteins across different species, with the inferred phylogenetic trees displayed on the left-hand side of the panel. AA residues altered in P1–P5 are annotated at the top of the figure and marked by red boxes. **(D)** Boxplot of transcriptomic distance to closest gnomAD variant for the de novo variants in P1–5 (blue) and gnomAD control variants (grey); *P = 0.02, Wilcoxon rank sum test. **(E)** Heatmap showing ΔΔG, a calculated parameter that predicts the effect of a protein change on stability, for selected mildly destabilizing ITPR3 variants across five different conformational models of ITPR3. A positive score (red) predicts that a variant is destabilizing, whilst a negative score (blue) predicts a variant to be stabilizing. Variants shown include those identified in P1–P5 of this study; V615M, a pathogenic dominant variant identified in familial CMT disease (Rönkkö et al., 2020); W168A, an in vitro generated variant that abolishes channel activity (Chan et al., 2010; Yamazaki et al., 2010); F1628L, a variant reported in a patient with later onset, milder immune deficiency (Neumann et al., 2023); P187S and I184S, two gnomAD control variants close to the variant detected in P1; and R2471H and I2511V, two further gnomAD control variants within the TMD close to the variants detected in P2–P5.

The four ITPR chains are held together by their N-terminal cytosolic segments, which harbor sites for binding IP_3_ and Ca^2+^, as well as by their C-terminal transmembrane domains (TMD), which form the Ca^2+^ channel (Fig. 3, A and B; interactive version at https://michelanglo.sgc.ox.ac.uk/r/itpr3). Binding of IP_3_ and Ca^2+^ trigger dynamic structural changes, which serve to transiently open the gate when the cytosolic Ca^2+^ concentration is low by inducing changes to the conformation of the TMD, including to a π-bulge centered around I2507 (Fan et al., 2018). In contrast, an inhibited, closed conformation is adopted in the presence of high cytosolic Ca^2+^ (Paknejad and Hite, 2018). The de novo ITPR3 variants identified in P1–P5 affect highly conserved AA residues, unlike the inherited R1850Q polymorphism additionally present in P2 (Fig. 3 C) and are located in regions of the *ITPR3* sequence relatively devoid of variants observed in healthy controls. The median exonic distance between the de novo variants found in P1–P5 and the closest variant reported in the gnomAD database of healthy controls is 17.5 base pairs versus six base pairs between gnomAD variants (P = 0.02, Wilcoxon rank sum test) (Fig. 3 D). The variant found in P1 (A196T) results in the substitution of a small hydrophobic alanine residue for a larger polar threonine residue in the coupling β-trefoil domain (BTF), responsible for controlling the conformation of the cytoplasmic portion of the complex via dynamic interchain interactions (Fig. 3, A and B, upper right inset). The de novo variants found in P2 (I2506N), P3 (R2524H), and P4–P5 (R2524C) are situated on the S6 helix of the TMD, flanking the gating residues (Fig. 3, A and B, middle and lower right insets). R2524H and R2524C disrupt interchain salt bridges normally formed by the arginine, and I2506N replaces a hydrophobic residue for a polar one on the wall of the ion channel near the gate. We explored the mechanisms by which the variants found in P1–P5 might impair IP_3_R function using in silico modeling, together with scrutiny of *ITPR3* variants reported elsewhere.

In the gnomAD database of healthy controls, we found one monoallelic start codon loss and 18 heterozygous truncating *ITPR3* variants (Fig. 3 A, red bars). The latter would either lead to nonsense-mediated decay or, if translated, result in shortened proteins predominantly lacking the TMD necessary for tetramerization and channel formation. Additionally, a homozygous frameshift variant was detected in a healthy individual in the GEL database, leading to the termination of the protein within the first quarter (data not shown). These observations suggest that for a heterozygous *ITPR3* variant to be pathogenic, it would need to be incorporated into the tetramer and interfere with the normal functioning of the channel. We thus next interrogated the possible structural and functional consequences of the *ITPR3* variants detected in P1–P5. The impact of each variant on protein stability was assessed for the various cryogenic electron microscopy (cryo-EM) structures available for study, representing the closed apoprotein, preactivated, activated, and inhibited conformations of IP_3_R3 (Paknejad and Hite, 2018). This was achieved by calculating the single state difference in Gibbs free energy, a proxy for thermodynamic stability, between the WT and variant protein conformations, expressed as ΔΔG. A negative score suggests a variant is stabilizing, whereas a positive score suggests that the variant is destabilizing with higher positive values predicting more deleterious changes (Gaspari, 2020). The de novo variants found in P1–P5 have in common an apparent favoring of the open channel conformation over the other forms, based on the predicted ΔΔG value being the lowest in the active state for all of them, with negative scores for the variants in P1, P2, and P3 (Fig. 3 E). This is in keeping with experimentally proven Ca^2+^ channel leakiness for R2524C and V615M, a neuropathy-causing variant (Rönkkö et al., 2020; Terry et al., 2022). However, this contrasts with an in vitro generated variant, W168A, that abolishes channel activity, and with F1628L, recently reported in combination with the R1850Q polymorphism in a patient with later onset immunodeficiency, which both mildly disfavor the open form and mildly favor closed conformations (Fig. 3 E) (Chan et al., 2010; Neumann et al., 2023; Yamazaki et al., 2010). R1850Q, the polymorphism inherited in P2, and T1424M, another neuropathy-causing variant experimentally shown to cause channel leakiness, could not be included in the heatmap as their residues have not been resolved in the required cryo-EM structures (Terry et al., 2022).

Finally, we also assessed missense *ITPR3* variants in the gnomAD database (Fig. 3 A, blue bars), with a focus on those located close to the variants detected in P1–P5. A total of 372 missense variants were found, 97 of which had an allele frequency higher than 5 × 10^−5^. Amongst these more frequent variants, six were predicted to be highly destabilizing (ΔΔG > 5 kcal/mol), further suggesting that, for ITPR3, pathogenicity is not a case of simple loss of protein stability. Two gnomAD variants, I184S and P187S, were found close to the cytosolic interface in proximity to A196T detected in P1. Both were predicted to be mildly destabilizing across most ITPR3 conformations and lack any relative favoring of the open conformation (Fig. 3 E), suggesting they may lack the dynamic, state-specific effect of A196T and indeed the other variants found in our cohort. 18 gnomAD variants were found within the TMD. Of these, three were found in the homozygous state (V2295M, I2273T, and E2398Q) and are predicted to be neutral. A further three heterozygous variants (Y2054C, L2114R, and R2262H) were very destabilizing (ΔΔG > 8 kcal/mol) and thus likely did not assemble, lending further evidence to reject haploinsufficiency as a pathogenic mechanism for ITPR3-related CID. Of the remaining 12 mildly destabilizing variants located in the TMD, two warranted further consideration: R2471H and L2511V (Fig. 3 E). R2471H was interesting, as although it disrupts salt bridging like the variants detected in P3–P5, it is located further from the gate (12 Å) than the variants observed in P2–P5 (3–7 Å), suggesting proximity to the gate is key for disrupting channel function for variants occurring within the TMD. It is further conceivable that the closeness of the variants in P2–P5 to residues important for inducing conformational changes within the TMD necessary to regulate channel opening, such as I2507, plays a role in their pathogenicity. There are no variants in the gnomAD database that are closer to the gate than the variants in P2–P5 (≤7 Å) except for L2511V, reported in two Finnish individuals, which lies 3 Å from the gate but is predicted to destabilize equally the open and closed conformation, and therefore is potentially neutral.

In conclusion, the de novo variants identified in P1–P5 affect three highly conserved AAs, suggesting that they are essential for the overall structure or function of ITPR3. The presence of truncations and strongly destabilizing missense variants in the gnomAD healthy control database in proximity to the patient variants suggests that haploinsufficiency is not the basis of pathogenicity. Furthermore, the presence of a healthy participant in GEL with a homozygous truncation means that a complete loss of ITPR3 is also tolerated. These observations support a more active role for the variants detected in P1–P5 in perturbing IP_3_R function. In support of this, in silico analyses suggest that incorporation of the patient protein variants into the IP_3_R complex may disrupt its function by favoring the open conformation of the channel relative to the closed or inhibited states by disrupting interactions in the neighborhood of the channel gate and/or by altering residues important for inducing functionally important conformational changes.

### Compromised Ca^2+^ signaling in patient T cells with depletion of ER Ca^2+^ stores likely due to IP_3_R channel leakiness

**Figure 4.**
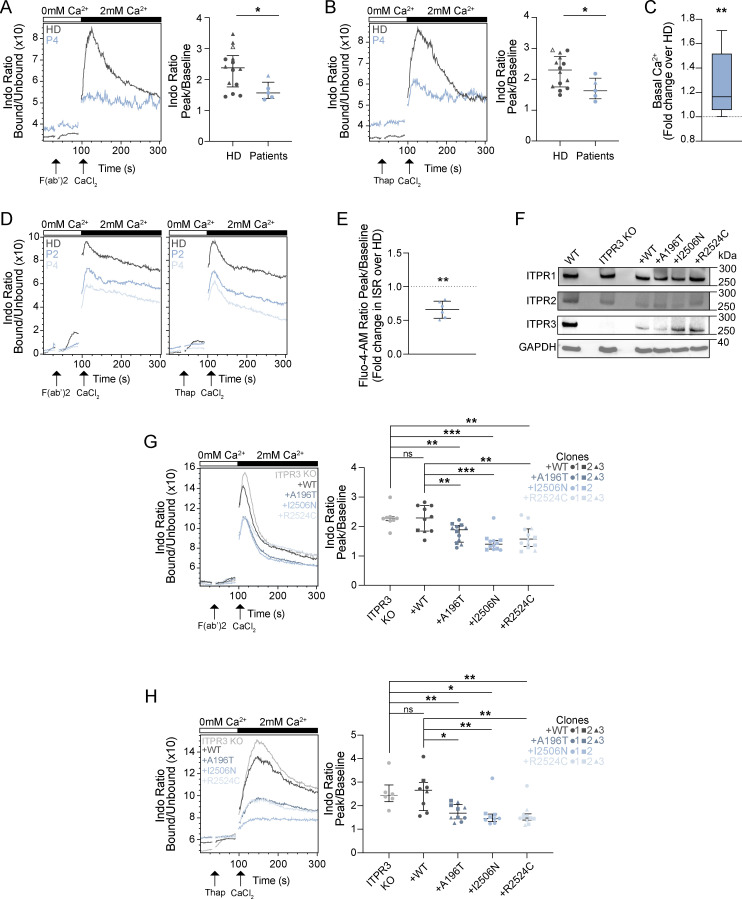
**Variant ITPR3 proteins impair Ca**^**2+**^ **flux in T** **cells. (A–C)** In primary T cells: Ca^2+^ flux after stimulation with (A) anti-CD3 plus F(ab′)2 and (B) thapsigargin (Thap) in Ca^2+^-free media and after addition of CaCl_2_. The left panels show representative examples of Ca^2+^ flux in primary T cells from P4 (blue) versus one HD (grey). The right panels show the ratio between the peak (100–150 s) versus baseline (0–30 s) in P2 (pre-HCT), P4 (untransplanted), and P5 (pre-HCT) versus 10 HDs. Independent replicates are included for two patients (*n* = 2 for P2 and P4) and three HDs. The lines show the median and the interquartile range. Triangles and circles distinguish between frozen and fresh samples, respectively (the HD indicated with an open triangle is the mother of P2, who carries the R1850Q polymorphism). *P < 0.05, Mann–Whitney U test. **(C)** Baseline cytosolic Ca^2+^ levels in primary patient T cells, expressed as fold change over HD. Data represent 12 HD and 12 patient samples (P2, P4, and P5), pooled across four independent experiments; **P <0.01, paired *T* test. **(D–E)** In CTLs: Ca^2+^ flux after stimulation with (D) anti-CD3/F(ab′)2 (left panel) and thapsigargin (right panel) in Ca^2+^-free media and after addition of CaCl_2_ in CTLs from P2 and P4 (blue) versus one HD (grey). This is representative of four independent experiments. **(E)** Ca^2+^ flux after stimulation with ionomycin in Ca^2+^-free media in CTLs from P2 and P4 (light blue) versus one HD; representative of two independent experiments. **P < 0.01, Welch’s *T* test. **(F–H)** In Jurkat T cell lines: (F) western blots for ITPR1, ITPR2, ITPR3, and GAPDH from protein extracts from WT Jurkat T cells, ITPR3 KO Jurkat T cells, and stably transduced ITPR3 KO Jurkat T cells expressing WT ITPR3 or the A196T, I2506N, and R2524C proteins; representative of two independent experiments. **(G–H)** The left panels show representative Ca^2+^ flux after stimulation with (G) anti-CD3 and F(ab’)2 or (H) thapsigargin in one ITPR3 KO Jurkat T cell clone (light grey) and in stably transduced ITPR3 KO Jurkat T cells (derived from the same ITPR3 KO clone) expressing WT ITPR3 (dark grey) or the A196T, I2506N, and R2524C protein variants (shades of blue). The right panel shows the ratio between the peak versus baseline in the ITPR3 KO clone and in transduced KO cells expressing WT (three clones) or variant ITPR3 proteins (three clones for A196T, two clones for I2506N, and three clones for R2524C) in five independent experiments. ns: non-significant, *P < 0.05, **P = 0.01, and ***P < 0.01, Mann–Whitney U test. Source data are available for this figure: SourceData F4.

To assess whether the patient ITPR3 protein variants adversely impact IP_3_R channel function, we investigated intracellular Ca^2+^ signaling by flow cytometry in primary T cells from four patients and CTLs generated from two patients using the fluorescent cytosolic Ca^2+^ indicators, Indo-1 and Fluo-4 AM (Fig. 4, A–E; Fig. S1, C–E; and Fig. 2 A). Following TCR stimulation, we could not capture ISR in primary T cells stimulated in Ca^2+^-free buffer. SOCE, measured upon addition of extracellular Ca^2+^, was significantly impaired in patient T cells in comparison to HD (P = 0.04, Mann–Whitney U test) (Fig. 4 A and Fig. S1 C). Peripheral blood mononuclear cells (PBMCs) were also treated with thapsigargin, which inhibits the sarco-/ER Ca^2+^-ATPase (SERCA) pump resulting in ER Ca^2+^ store depletion and subsequent SOCE independently of the IP_3_R (Gouy et al., 1990). Compared with HD T cells, after thapsigargin, we again observed reduced SOCE in patient T cells (P = 0.04, Mann–Whitney U test) (Fig. 4 B), reflecting their generally altered Ca^2+^ homeostasis. SOCE was also reduced in primary patient T cells compared with HD T cells after stimulation in Ca^2+^-containing media (Fig. S1 D and data not shown). Later addition of ionomycin, a membrane-permeable Ca^2+^ ionophore which besides mobilizing intracellular Ca^2+^ stores also facilitates Ca^2+^ influx directly across the plasma membrane (Nüsse et al., 1997; Putney, 2010), resulted in cytosolic Ca^2+^ increase comparable with HD (Fig. S1 D). Given the predictions from the in silico modeling above, we speculated that the blunted SOCE observed after thapsigargin could be due to already depleted ER Ca^2+^ stores in patient T cells at baseline due to IP_3_R channel leakiness. Channel leakiness was recently shown as the disease-causing mechanism in CMT patients with missense *ITPR3* variants, including for the CID-associated R2524C variant (Terry et al., 2022). In line with this, cytosolic (Ca^2+^) was higher in patient primary T cells compared with HD T cells at baseline, in the absence of stimulation and extracellular Ca^2+^ (Fig. 4 C). Whilst higher baseline cytosolic (Ca^2+^) was not consistently detected by flow cytometry in CTLs generated for P2 and P4, ISR could be captured in HD CTLs after TCR stimulation, thapsigargin and ionomycin, but appeared negligible in patient-derived CTLs, further suggesting depletion of ER Ca^2+^ stores (Fig. 4 D and Fig. 1 E). To further quantify ER Ca^2+^ stores in patients versus HD CTLs, cells were stimulated with ionomycin in Ca^2+^-free media, and ISR was quantified by computing the ratio of peak/baseline Fluo-4 AM fluorescence. In comparison with HD, the rise in cytoplasmic Ca^2+^ was significantly blunted in patient-derived CTLs (P = 0.0012, Welch’s *t* test), consistent with depletion of intracellular Ca^2+^ stores in patient cells (Fig. 4 E). Consistent with the results obtained in primary T cells, following provision of extracellular Ca^2+^, SOCE was impaired in patient CTLs after TCR stimulation and thapsigargin but comparable with HD after ionomycin (Fig. 4 D and Fig. S1 E). No significant changes in cytosolic Ca^2+^ dynamics were observed in patients’ fibroblasts when compared with cells of healthy controls (Fig. S1 F), suggesting cell-type specific differences or compensations.

Overall, these results show that, in patient T cells, the ITPR3 protein variants affect the receptor’s channel function with altered Ca^2+^ homeostasis and blunted Ca^2+^ dynamics after stimulation. The elevated baseline cytosolic (Ca^2+^) in patient primary T cells and blunted ISR in patient-derived CTLs indicates that this is due to IP_3_R channel leakiness with subsequent depletion of ER Ca^2+^ stores.

### ITPR3 protein variants are responsible for the dominant negative disruption of IP_3_R function

To confirm that the patient variants are directly responsible for impaired Ca^2+^ signaling in T cells, and to further investigate their pathogenic mechanisms, we generated gene-edited Jurkat T cell lines. We first knocked out *ITPR3* (ITPR3 KO) using CRISPR/Cas9 electroporation of WT Jurkat T cells, and then, via lentiviral transduction of one KO clone, generated multiple Jurkat T cell clones stably expressing similar quantities of either WT ITPR3 protein or one of the patient ITPR3 protein variants (A196T, I2506N, and R2524C) (Fig. 4 F). When assessing Ca^2+^ signaling by flow cytometry, we found that WT Jurkat T cells, ITPR3 KO clones, and those derived from reintroducing WT ITPR3, had similar SOCE in response to TCR stimulation and thapsigargin (Fig. 4, G and H; and Fig. S1 G), confirming the in silico predictions that impaired Ca^2+^ responses in patient T cells are not due to *ITPR3* haploinsufficiency. In fact, complete loss of ITPR3 was tolerated, likely due to functional redundancy with ITPR1/2. In contrast, SOCE was impaired when the patient ITPR3 protein variants were re-expressed in KO cells after both TCR stimulation and thapsigargin (Fig. 4, G and H). These findings demonstrate that the variant ITPR3 proteins are directly responsible for the defective SOCE observed in patient-derived T cells.

IP_3_R channel leakiness and depletion of intracellular Ca^2+^ stores could theoretically result in constitutively active SOCE. To further investigate this, we measured cytosolic Ca^2+^ influx upon the addition of extracellular Ca^2+^ but without prior agonist stimulation to mobilize intracellular Ca^2+^ stores. In ITPR3 KO Jurkat T cell clones expressing WT or variant ITPR3 proteins, there was a small increase in intracellular Ca^2+^ after the provision of extracellular Ca^2+^ (Fig. S1 H); however, this was lower than the Ca^2+^ influx measured after agonist stimulation (Fig. S1 HI) and was not increased in cells expressing the variant ITPR3 proteins versus WT (Fig. S1 HII). A similar result was found in CTLs generated from P2 and P4 (Fig. 1 I). These findings are not consistent with constitutive SOCE in patient T cells.

Overall, we concluded that the de novo missense *ITPR3* variants in P1–P5 result in the expression of dominant-negative proteins that interfere with the function of IP_3_R heterotetramers by causing channel leakiness and depletion of intracellular Ca^2+^ stores, resulting in impaired SOCE in T cells.

### Patients with *ITPR3* variants have T cell lymphopenia with phenotypic features of activation and exhaustion

**Figure 5.**
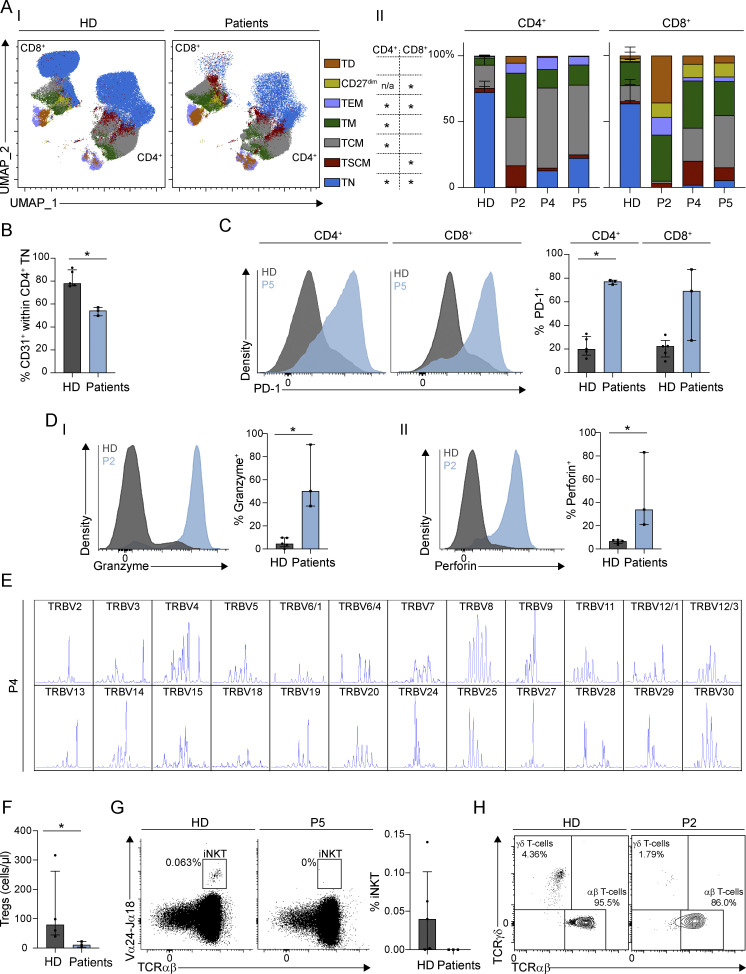
**T** **cell phenotype in non-transplanted patients with heterozygous *ITPR3* variants. (A)** Distribution of maturation-associated T cell subsets in patients (P2, P4, and P5) versus three pediatric HDs displayed as (I) UMAP projections in concatenated HD (left) and patient (right) samples and (II) stacked bar charts for CD4^+^ (left) and CD8^+^ T cells (right); HD data has been combined and mean and standard deviation (error bars) are plotted. Statistical significance is displayed in the chart in the center of the figure; *P < 0.05, Mann–Whitney U test, across three independent experiments. Maturation-associated subsets are colored according to phenotype and include naïve (TN: CD45RA^+^CD27^+^CCR7^+^CD95^−^; blue), stem central memory (TSCM: CD45RA^+^CD27^+^CCR7^+^CD95^+^; red), central memory (TCM: CD45RA^−^CD27^+^CCR7^+^CD95^+^; grey), transitional memory (TM: CD45RA^−^CD27^+^CCR7^−^CD95^+^; dark green), effector memory (TEM: CD45RA^−^ CD27^−^CCR7^−^CD95^+^; lilac), and terminally differentiated (TD: CD45RA^+^CD27^−^CCR7^−^CD95^+^; orange) CD4^+^ and CD8^+^ T cells, as well as effector CD27^dim^ (CD45RA^+^CD27^dim^CCR7^−^CD95^+^; light green) CD8^+^ T cells. **(B)** Bar chart showing the percentage of CD31^+^ RTEs within naïve CD4^+^ T cells in patients versus pediatric HDs. **(C)** Bar chart (right) showing the percentage of PD-1^+^ cells in CD4^+^ and CD8^+^ T cells in patients versus pediatric HDs; representative histograms (left) are shown for P5 and one HD. **(D)** Bar chart (right) showing the percentage of CD8^+^ T cells positive for (I) granzyme B and (II) perforin in patients versus pediatric HDs. Representative histograms (left) are shown for P2 and one HD. **(E)** Representative TCRVβ spectratyping shown for P4 on isolated CD3^+^ T cells. All Vβ families are represented; all but one (TRBV30) have a skewed and sparse profile with oligoclonal expansions present and a median number of seven peaks. **(F)** Bar chart showing absolute numbers of Tregs in patients versus pediatric HDs. **(G)** Bar chart (right) showing the percentage of iNKT cells within T cells in patients versus pediatric HDs. Representative flow cytometry plots (left) for P5 and one HD are displayed. **(H)** Representative flow cytometry plots for P2 and one HD showing the percentage of TCR γδ^+^ and ⍺β^+^ cells with total CD3^+^ T cells. For B–D and F–G, representative of three independent experiments, bars represent the median values and lines and the interquartile range. *P < 0.05, Mann–Whitney U test. All statistics were performed using bilateral *t* tests. n/a, not available.

**Figure S2.**
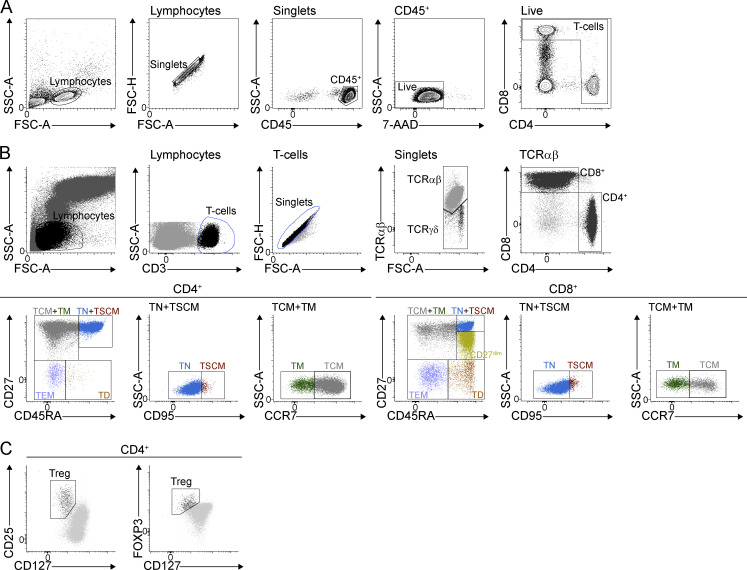
**Gating strategies for flow cytometric analyses and sorting. (A)** FACS gating employed for (A) ratiometric analysis of cytoplasmic Ca^2+^ flux in primary T cells. **(B)** Immunophenotyping of T cells. TN: naïve; TSCM: stem cell memory; TCM: central memory; TM: transitional memory; TEM: effector memory; TD: terminally differentiated; CD27^dim^: effector CD27^dim^. **(C)** Enumeration of Tregs.

To investigate the relevance of impaired lymphocyte Ca^2+^ signaling, we completed a comprehensive immunological assessment of three patients (P2 before HCT, P4, and P5 before chemotherapy). All were T cell lymphopenic and showed striking phenotypic alterations within their T cell compartment (Fig. 5 and Table S1). We determined the frequencies of CD4^+^ and CD8^+^ T cell subpopulations and compared them with age-matched controls (Fig. 5 A and Fig. S2 B). We observed a marked reduction in the absolute numbers and proportions of naïve T cells (P = 0.03, Mann–Whitney U test) (Fig. 5 A and Table S1) and noted a decreased frequency of CD31^+^ recent thymic emigrants (RTEs) amongst naïve CD4^+^ T cells (P = 0.03, Mann–Whitney U test) (Fig. 5 B). Conversely, the proportion of T cells with a memory phenotype was increased in the patients (Fig. 5 A). Within CD8^+^ T cells, cells with a terminally differentiated phenotype were expanded (P = 0.05, Mann–Whitney U test) (Fig. 5 A). Additionally, there was increased expression of inhibitory receptors PD-1 (P = 0.05 for CD8^+^, P = 0.03 for CD4^+^, Mann–Whitney U test) (Fig. 5 C) and 2B4 (Table S1), which are markers of exhaustion. Though, as these analyses were performed on total CD4^+^ and CD8^+^ T cells, the lack of naïve cells and skewing toward effector and memory cells in the patients may, at least in part, account for these findings. Patient CD8^+^ T cells concurrently also expressed markers associated with activation, including HLA-DR (Table S1) and the granule components, granzyme B and perforin (P = 0.03, Mann–Whitney U test) (Fig. 5 D). TCR repertoire analysis by CDR3 spectra typing identified all TCRVβ families, but expression patterns were abnormal with oligoclonal expansions (Fig. 5 E and Table 2).

Because reduced cell counts have been reported in patients with abolished SOCE and in mouse models of CRAC channelopathies, we assessed the numbers of T regulatory cells (Treg) and invariant NK T cells (iNKT). Absolute numbers of Treg were reduced (P = 0.03, Mann–Whitney U test) and iNKT cells could not be detected (P = 0.06, Mann–Whitney U test) in patients (Fig. 5, F and G; and Table S1). γδ T cells were profoundly reduced in P2 and P5 pre-HCT, as well as in P4 (Fig. 5 H and Table S1).

In summary, patients with *ITPR3* variants display changes in the distribution of T cell subsets, with phenotypic features of both activation and exhaustion, and oligoclonal T cell repertoires.

### IP_3_-mediated Ca^2+^ signaling plays a role in late stages of T cell development

**Figure 6.**
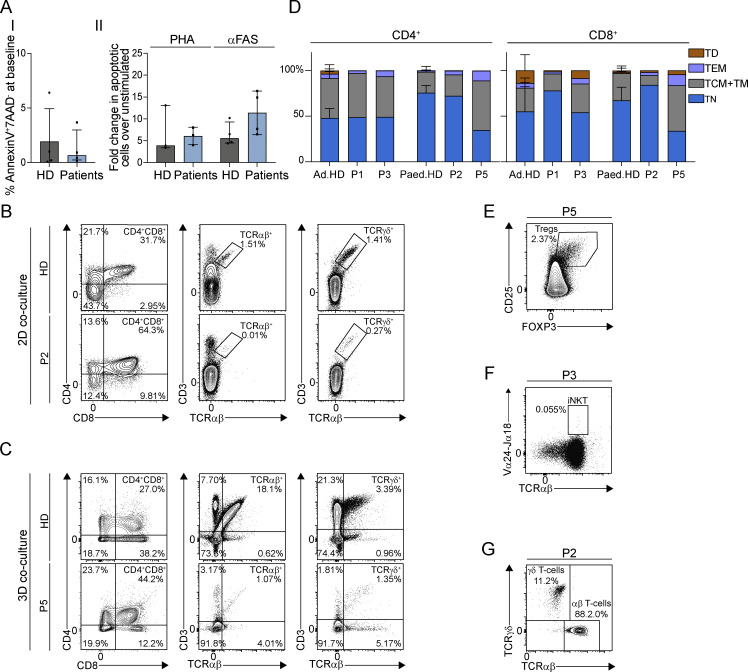
**Heterozygous *ITPR3* variants primarily cause a hematopoietic defect correctable via HCT. (A)** Graphs showing percentage of PBMCs undergoing apoptosis (AnnexinV^+^7AAD^−^) in patients (P2: two to three independent replicates; P4: one replicate) versus HDs at (I) baseline, representing apoptosis determined on day 0 in unstimulated PBMCs; this is shown alongside (II) fold change in percentage apoptotic cells in stimulated versus unstimulated PBMCs after culture with anti-CD3 and IL-2 for 6–7 days, followed by stimulation with PHA or anti-FAS IgM. Bars represent the media and lines the interquartile range; results are not statistically significant via multiple unpaired *t* tests with Holm–Sidak correction for multiple comparisons. **(B and C)** Flow cytometry plots showing proportions (within live CD45^+^ cells) of CD4^−^CD8^−^ double negative (DN) T cells, CD4^+^CD8^+^ DP T cells, CD3^+^TCRαβ^+^, and CD3^+^TCRγδ^+^ T cells (B) after 6 wk of ex vivo T cell differentiation of CD34^+^ cells isolated from P2 (bone marrow) and from one HD (cord blood) in a two-dimensional (2D) co-culture with OP9/DL1 stromal cells, and **(C)** after 6 wk of differentiation of CD34^+^ cells isolated from P5 (bone marrow) and from one HD (cord blood) in a three-dimensional (3D) co-culture with MS5/DL1 stromal cells in ATOs. All co-cultures were set up with at least three replicates in each experiment. **(D)** Distribution of maturation-associated T cells subsets displayed in stacked bar charts for CD4^+^ (left) and CD8^+^ T -cells (right) in P1, P2, P3, and P5 post-HCT at last follow-up versus adult (Ad.) and pediatric (Ped.) HDs; HD data has been combined and mean and standard deviation (error bars) are plotted. **(E–G)** Representative flow cytometry plots showing recovery of (E) Treg, (F) NKT, and (G) γδ T cells in patients post-HCT; representative of two to three independent experiments.

In contrast to the typically normal T cell counts in CRAC channelopathies (Badran et al., 2016; Byun et al., 2010; Feske et al., 2006, 2010; Fuchs et al., 2012; Lacruz and Feske, 2015; Lian et al., 2018; McCarl et al., 2009; Picard et al., 2009; Schaballie et al., 2015; Yu et al., 2021), ITPR3 patients have peripheral T cell lymphopenia alongside markers of poor thymic output including negligible RTEs and TREC levels (Table 2 and Fig. 5 B). We found comparable proportions of apoptotic cells (AnnexinV^+^7AAD^−^) in patients versus HD PBMCs, either at baseline in the absence of stimulation, or after culture with anti-CD3 and IL-2 for 6–7 days, followed by stimulation with PHA or anti-FAS IgM (Fig. 6 A). This led us to consider whether impaired IP_3_-mediated signaling and/or impaired Ca^2+^ homeostasis could directly affect T cell development. To assess whether patient hematopoietic stem and progenitor cells (HSPCs) are intrinsically able to commit to the T cell lineage and undergo initial stages of intrathymic development, we co-cultured CD34^+^ HSPCs from P2 and P5 with genetically modified murine stromal cells expressing the human Notch ligand Delta-like 1 in a two-dimensional assay (P2) (Schmitt and Zúñiga-Pflücker, 2002; Six et al., 2011) or a three-dimensional artificial thymic organoid (ATO) assay (P5) (Seet et al., 2017), and assessed for evidence of T-lineage commitment and differentiation ex vivo. After 6 wk of co-culture, there was an accumulation of CD4^+^CD8^+^ double positive (DP) T cells in patients compared with HD differentiated cells (Fig. 6, B and C). Differentiated patient-derived TCRαβ- and TCRγδ-expressing CD3^+^ T cells could also be detected, but in lower proportions than for HDs (Fig. 6, B and C). Additionally, ex vivo differentiated patient CD4^+^ and CD8^+^ T cells showed increased basal cytosolic Ca^2+^ levels and severely impaired SOCE compared with differentiated HD T cells after TCR stimulation (Fig. 1 J) and after thapsigargin treatment (data not shown). This suggests that, at least ex vivo, ITPR3 variant HSPCs are not intrinsically impaired in their ability to give rise to early T-lineage DP lymphocytes, but that IP_3_-mediated signaling specifically plays a role in later stages of T cell development. Likewise, histopathological assessment of a thymus biopsy from P1 showed morphologically normal thymic tissue with no lymphodepletion (data not shown).

Four out of five patients included in this study have undergone HCT. P1, P2, and P5 were transplanted in childhood for significant, symptomatic CID, and P3 was transplanted in early adulthood after developing lymphoma. P1, P2, P3, and P5 are now 14, 2, 9, and 0.5 years after HCT with 41%, 100%, 91%, and 100% donor T cell engraftment, respectively (Table 2). As expected, T cell Ca^2+^ signaling in response to CD3 stimulation and thapsigargin is normal after HCT (tested in P2 and P3; data not shown). At the last follow-up, P1–P3 were clinically well with reduced infection susceptibility, normal T cell counts, proliferative responses to PHA, and spectratyping (Table 2). Furthermore, they now have significant TREC levels (Table 2) and display normal proportions of naïve T cells (Fig. 6 D) with the recovery of Treg, iNKT, and γδ T cells (Fig. 6, E–G). Though P5 was only recently transplanted, it showed early signs of successful immune reconstitution with increasing naïve T cell counts (Fig. 6 D).

### Abnormal Ca^2+^ signaling is associated with partially impaired function of ITPR3 variant T cells

**Figure 7.**
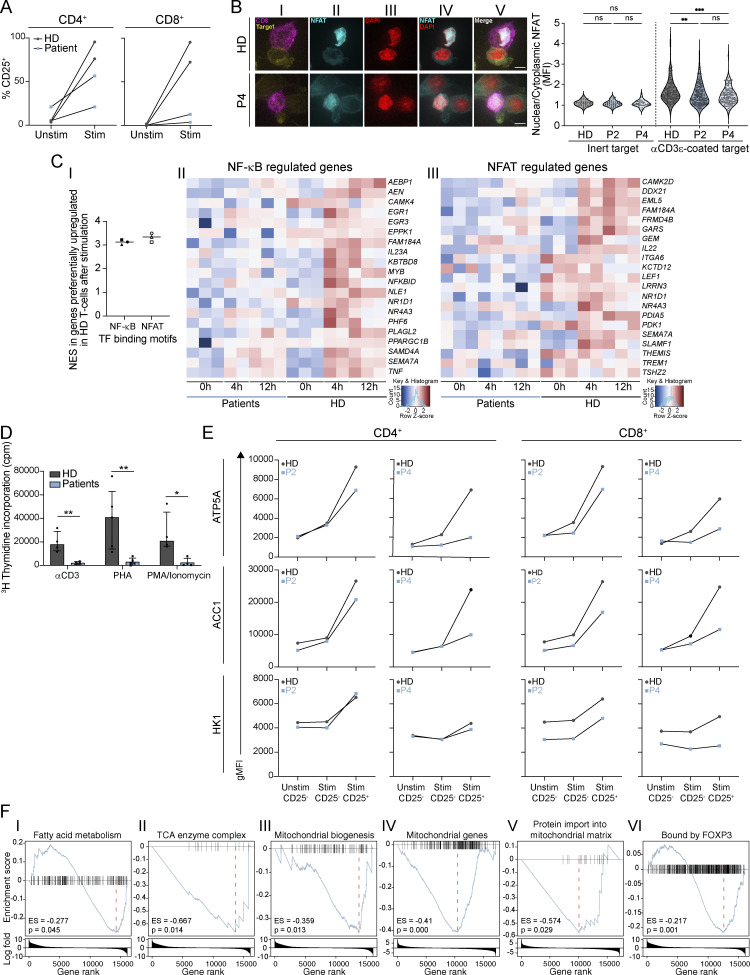
**T** **cells with heterozygous *ITPR3* variants are impaired in their ability to express NF-κB and NFAT-dependent genes, proliferate, and upregulate metabolic enzymes in response to stimulation. (A)** Graphs showing the percentage of CD25^+^ cells in CD4^+^ (left) and CD8^+^ T cells (right) stimulated with anti-CD3/CD28 compared with unstimulated for P2, P4, and two HDs across two independent experiments. **(B)** Nuclear translocation of NFAT after TCR engagement in CTLs from P2, P4, and two HDs in three independent experiments. The left panel shows representative images of (I) P185 target cells (yellow) and stimulated CD8^+^ CTLs (purple), (II–IV) intracellular staining for NFAT (blue) and nuclear staining with DAPI (red), and (V) a composite image illustrating the mildly reduced nuclear NFAT translocation in P4 compared to HD; scale bar = 5 μm. The right panels show the ratios between nuclear NFAT and cytoplasmic NFAT measured in MFI in unstimulated and stimulated CTLs from P2, P4 and the HDs in all three experiments; ns, non-significant, **P <0.01 and ***P <0.001, one-way ANOVA with Šídàk’s multiple comparisons test. **(C)** Genes activated by NF-κB and NFAT are downregulated in patient T cells compared with HD following anti-CD3/CD28 stimulation for 4 or 12 h (I) Graph showing normalized enrichment scores (NES) for NF-κB and NFAT transcription factor (TF) binding motifs within 5 Kb of the transcriptional start sites of genes significantly upregulated in HD versus patient T cells after 4 h of stimulation; filled symbols denote NF-κB motif analysis: square=bergman_dif_Rel, circle=homer_GGGGGAATCCCC_NFkB-p50_p52, triangle=transfac_pro_M01223; empty symbols denote NFAT motif analysis: square=taipale_NFATC1_full_NATGGAAANWWWWTTTYCMN_repr, circle=taipale_NFATC1_full_TTTTCCATGGAAAA_repr, triangle=cisbp_M5658. (II and III) Heatmaps showing expression level of genes significantly upregulated in HD versus patient T cells following stimulation that contain TF binding motifs for (II) NF-κB and (III) NFAT. **(D)** Bar chart showing proliferation of PBMCs from P1 (pre-HCT), P2 (pre-HCT), P4, and P5 (pre-HCT) versus HDs after stimulation with anti-CD3, PHA, or PMA and ionomycin. Proliferation was assessed by measuring the incorporation of titrated (^3^H) thymidine and quantified as counts per minute (cpm) over background across six independent experiments. Bars represent the median values and lines the interquartile range; *P < 0.05, **P < 0.01, *t* test with Sidak-Bonferonni correction for multiple comparisons. **(E)** Graphs showing expression (gMFI) of metabolic enzymes in CD4^+^ (left) and CD8^+^ T cells (right) stimulated with anti-CD3/CD28 compared with unstimulated cells for P2, P4, and two HDs across two independent experiments. **(F)** Gene set enrichment analyses of differentially expressed genes identified by RNA-sequencing of patients (P2, P4, and P5) and HD T cells (*n* = 3) stimulated with anti-CD3/28. Enrichment scores (ES) and P values are displayed for the following gene sets: (I) fatty acid metabolism, (II) tricyclic acid (TCA) enzyme complex, (III) mitochondrial biogenesis, (IV) mitochondrial genes, (V) protein import into the mitochondrial matrix, and (VI) bound by FOXP3.

**Figure S3.**
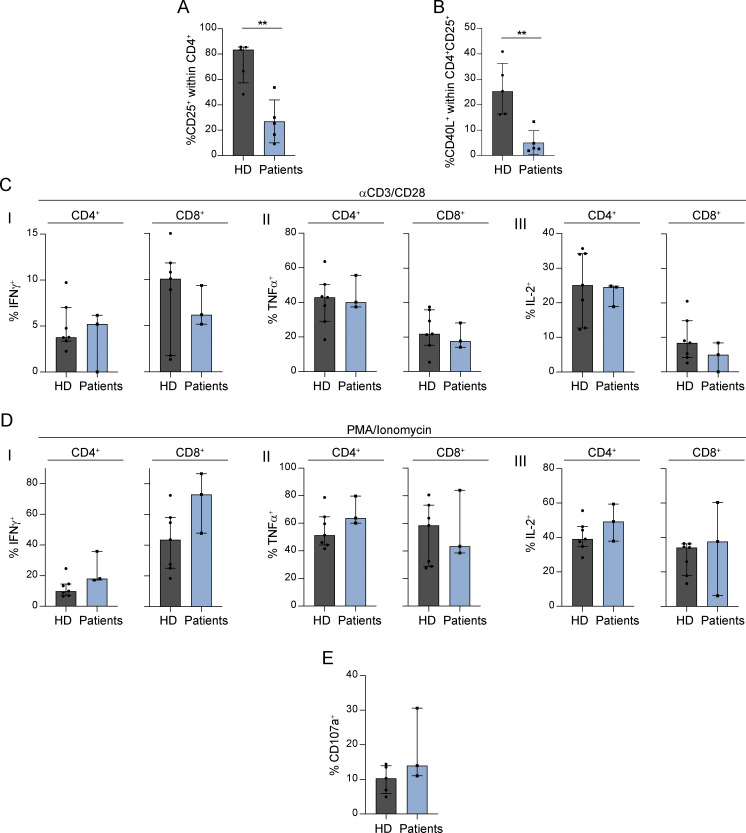
**Functional T** **cell assays in patients with heterozygous *ITPR3* variants. (A and B)** Graphs showing the percentage of (A) CD25^+^ cells within CD4^+^ T cells and (B) CD40L^+^ cells within activated CD25^+^ CD4^+^ T cells after stimulation with PHA in patients (P2: three replicates; P4: two replicates) versus HDs, across three independent experiments; **P < 0.01, two-tailed *t* test. **(C and D)** Graphs showing interferon γ (IFNγ), tumor necrosis factor α (TNFα), and interleukin (IL)-2 production in patients (P2, P4, and P5) versus HD T cells after stimulation with (C) anti-CD3/28 and (D) PMA/Ionomycin. Results are expressed as a percentage of cytokine^+^ cells within CD4^+^ and CD8^+^ T cells after excluding naïve cells. **(E)** Degranulation capacity of CD8^+^ T cells after stimulation with anti-CD3/CD28. Results are expressed as a percentage of CD107a^+^ cells within total CD8^+^ T cells, across three independent experiments, two-tailed *t* test.

Given the impaired Ca^2+^ signaling in T cells from patients with heterozygous missense variants in *ITPR3*, we also set out to determine the impact on T cell function. As SOCE controls NF-κB and NFAT activity in lymphocytes by promoting their nuclear translocation, we specifically investigated these two pathways. Using either anti-CD3/CD28 or PHA for primary T cell stimulation, we showed impaired upregulation of CD25 and CD40L, both transcriptional targets of NF-κB (Fig. 7 A; and Fig. 3, A and B). Subtly impaired nuclear translocation of NFAT1 was also observed by confocal microscopy in CTLs derived from P2 and P4 compared to HD in response to TCR stimulation (P = 0.0064 and P = 0.0004 respectively, ordinary one-way ANOVA with Holm-Šídák’s multiple comparisons test) (Fig. 7 B). We further looked for differential activation of the NF-κB and NFAT signaling pathways in the transcriptomes of patients versus HD primary T cells upon stimulation with anti-CD3/CD28. We found that upstream NF-κB and NFAT transcription factor binding motifs were enriched only amongst those genes upregulated in HD samples relative to patients following stimulation (Fig. 7 C). Transcripts for *IL2* and *IFNG* were significantly reduced in patients relative to controls after stimulation (*IL2*: log_2_ fold change = −2.81, P = 2.33e−15, FDR = 3.75e−11; *IFNG* log_2_ fold change = −1.35, P = 7.36e−5, FDR = 0.01). However, when assessed at the protein level, production of IL-2, IFNγ, and several other cytokines (IL-4, IL-7, TNFα) after mitogenic stimulation was comparable between patients and HDs (Fig. S3, C and D and data not shown). Similarly, the degranulation capacity of patient CD8^+^ T cells after CD3/CD28 stimulation was not impaired (Fig. S3 E). However, proliferative responses were severely abrogated in patient cells stimulated with anti-CD3, PHA, and PMA/ionomycin in comparison with HDs (Fig. 7 D). Cell growth and proliferation following TCR stimulation require metabolic reprogramming to meet increased energetic demands (Wang et al., 2020). This is in part regulated by Ca^2+^ signaling (Vaeth et al., 2017; Wang et al., 2020). We thus sought to determine whether defective metabolism could underlie the inability of patient T cells to proliferate. Using a flow cytometric assay (Ahl et al., 2020), we found that patient CD8^+^ T cells were impaired in their capacity to upregulate ATP5A and acetyl Co-A carboxylase 1 (ACC1), enzymes involved in mitochondrial ATP and fatty acid biosynthesis, respectively (Ahl et al., 2020), in response to stimulation (Fig. 7 E). Moreover, at baseline as well as after TCR stimulation, patient CD8^+^ T cells displayed a reduced expression of hexokinase 1 (HK1), an enzyme that couples cytosolic glycolysis to intramitochondrial oxidative phosphorylation (OXPHOS) (Fig. 7 E) (Ahl et al., 2020; Bantug et al., 2018). To further investigate the pathways and processes responsible for the patients’ impaired responses to stimulation, we performed gene set enrichment analyses of genes differentially expressed between the patient and HD T cells after stimulation with anti-CD3/CD28. This revealed the downregulation of multiple metabolic and mitochondrial pathways in patient T cells relating to fatty acid metabolism, the tricarboxylic acid (TCA) cycle, mitochondrial biogenesis, mitochondrial gene expression, and protein import into the mitochondrial matrix; FOXP3 target genes were also significantly downregulated in patient T cells after stimulation (Fig. 7 F).

Altogether, these results confirm that ITPR3-regulated Ca^2+^ dynamics are critical for certain key T cell functions, such as those driven by proper NF-κB and NFAT activation, and proliferative and metabolic responses. This offers further important insights into the mechanisms by which Ca^2+^ operates as a second messenger regulating multiple T cell functions.

## Discussion

We identified four de novo missense variants in *ITPR3* in five unrelated patients with childhood-onset CID and profound T cell lymphopenia (Fig. 1 and Fig. 2). *ITPR3* encodes a subunit of the IP_3_R, which functions as a ligand-gated Ca^2+^ channel in the ER membrane (Fan et al., 2015, 2018). Channel opening and depletion of ER Ca^2+^ stores activate STIM and ORAI proteins, inducing a massive influx of extracellular Ca^2+^, which activates intracellular enzymes and transcription factors that regulate T -cell functions. The critical importance of this pathway for T cells is demonstrated by defects that impair lymphocyte Ca^2+^ signaling (Bousfiha et al., 2020). Abolished SOCE in patients with biallelic LOF variants in *STIM1* or *ORAI1* variably impairs T cell function, often causing early-onset CID necessitating HCT (Badran et al., 2016; Byun et al., 2010; Feske et al., 2006, 2010; Fuchs et al., 2012; Lacruz and Feske, 2015; Lian et al., 2018; McCarl et al., 2009; Picard et al., 2009; Yu et al., 2021). Whereas severely reduced SOCE in a patient with compound heterozygote variants in CRAC channel regulator 2 A (CRACR2A), a protein that stabilizes STIM1 and ORAI1 interactions, has been shown to underlie late-onset CID (Rice et al., 2021; Srikanth et al., 2010). Recently, compound heterozygous variants in *ITPR3* have been reported in two patients with CID of variable onset and severity (Neumann et al., 2023). Whilst demonstrating a first link between immunodeficiency and IP_3_R dysfunction, the investigation of this novel Ca^2+^ channelopathy remained limited as only one of the compound heterozygous patients was not transplanted. Further expanding upon this report, we showed defective T cell Ca^2+^ signaling in five patients with early onset CID and monoallelic missense *ITPR3* variants actively disrupting IP_3_R channel function (Fig. 4 and Fig. S1). In-depth immunophenotyping and functional characterization (Fig. 5, Fig. 6, and Fig. 7), together with compelling clinical overlap with the non-immunological features seen in STIM1/ORAI1 channelopathies, such as ED and peripheral neuropathy, establish monoallelic missense variants in *ITPR3* as a cause of syndromic CID.

IP_3_R channel dysfunction has been associated with several disorders, such as spinocerebellar ataxia, Gillepsie syndrome, and generalized anhidrosis, mainly due to variants in *ITPR1* and *ITPR2* (Kerkhofs et al., 2018; Terry et al., 2018). To date, monoallelic missense variants in *ITPR3* have only been described in CMT disease (Rönkkö et al., 2020). One variant (V615M) was found in a family with adult-onset CMT, but neither immunodeficiency nor ED was reported despite long-term follow-up. CID, certainly of the severity seen in P1–P5 in this study, seems unlikely. An unrelated patient with early-onset CMT, who has been lost to follow up, had the same de novo variant (R2524C) that we found in P4 and P5. It is possible that immune deficiency had not been recognized in this patient or had not yet evolved. CID has only recently been linked to ITPR3 defects in two unrelated patients (Neumann et al., 2023). One patient also had a de novo R2524C variant and presented with early onset CID necessitating HCT, as well as CMT and ED. The second inherited an F1628L substitution from an asymptomatic parent and displayed a milder, later-onset immunodeficiency phenotype, without neuropathy or ED. A compound heterozygous basis was suggested as both patients additionally carried a second *ITPR3* variant, R1850Q. However, this variant does not affect a conserved residue (Fig. 3 C), has an allele frequency of 8.2% in gnomAD controls, including 6,079 homozygotes, and in both cases, had been inherited from clinically unaffected parents. P2 from this study also inherited the R1850Q polymorphism from her mother, who was clinically unaffected and had normal Ca^2+^ flux in T cells (Fig. 4, A and B, open triangle within HD data), and P3–5 had changes at the R2524 AA position without any additional *ITPR3* variants. Hence, we conclude that R1850Q is unlikely to be of great biological consequence and does not preclude dominant disruption caused by the rare de novo variants in P1–5. The inherited F1628L variant in the previously reported patient with late-onset CID displayed different characteristics in the in silico modeling compared with the de novo variants (Fig. 3 E), with relative favoring of the closed, preactive form rather than the open channel conformation. It is therefore possible that the molecular mechanism is different for this patient whose milder disease may be dependent on the coinheritance of two heterozygous variants, which are insufficient to cause disease in isolation. It is also possible that the R1850Q polymorphism plays a modifying role, including in P2 whose more severe immunodeficiency was apparent in infancy. Nonetheless, it is likely that variable penetrance and expressivity is a feature of *ITPR3*-related disease, as is the case in many IEIs, including STIM1 deficiency (Parry et al., 2016; Rice et al., 2019; Schaballie et al., 2015). In line with this, only three out of five patients in our cohort required HCT during childhood, four displayed variable features of ED, and just two were diagnosed with peripheral neuropathy to date (Table 1). Recognition and careful longitudinal description of more cases in the future are necessary to achieve a better appreciation of disease expression and genotype–phenotype correlations.

How variant IP_3_Rs affect channel function remains poorly understood. Most diseases associated with IP_3_R dysfunction display autosomal dominant inheritance, suggesting that the IP_3_R channel is most likely assembled from a combination of WT and variant IP_3_R monomers (Foskett, 2010; Huang et al., 2012; Rönkkö et al., 2020). Investigating the molecular consequences of variant IP_3_R3s in primary cells is therefore possibly complicated by partial compensation from endogenous WT IP_3_R1 and IP_3_R2 isoforms. By widening in silico modeling to *ITPR3* variants identified in healthy individuals in gnomAD and GEL (Fig. 3), we nevertheless excluded haploinsufficiency as the mechanism for disease and confirmed this by demonstrating unchanged SOCE in ITPR3 KO T cell lines (Fig. 4). By expressing the de novo ITPR3 variants in these KO T cells, we further proved their pathogenicity and concludes that rather than haploinsufficiency, the Ca^2+^ defect is due to the incorporation of abnormal IP_3_R3 subunits into the heterotetramer complex, actively altering its channel functions. This is in line with the findings from a recent study investigating the impact of disease-associated heterozygous missense *ITPR1* and *ITPR2* variants using concatenated IP_3_R constructs assembling into defined heterotetramers upon transfection into *ITPR1*^*−/−*^*ITPR2*^*−/−*^*ITPR3*^*−/−*^ triple KO (TKO) HEK cells (Terry et al., 2020). They indeed report that the stoichiometric arrangement of the variant and WT subunits within the receptor complex contributes to IP_3_R channel dysfunction.

All functional domains of IP_3_Rs are affected by disease-causing *ITPR* missense variants (Kerkhofs et al., 2018; Terry et al., 2018). Depending on the exact location of the variant, IP_3_R dysfunction and disease pathogenesis can result from the disturbance of multiple molecular mechanisms, such as IP_3_ binding, receptor stability and localization, channel permeation, or allosteric receptor regulation of the variant (Terry et al., 2020). The *ITPR3* variants detected in P1–P5 affect three highly conserved AAs, occurring in regions important for inducing conformational changes that regulate channel opening/closing (Fig. 3). In theory, the diminished SOCE we observed in patient T cells after IP_3_-mediated signaling could either occur via a dominant negative effect, resulting in reduced ISR of Ca^2+^, or a gain of channel function, resulting in a constitutively open or leaky channel. In silico modeling favored the latter, as did the observation of absent ISR in stimulated patient-derived CTLs and reduced SOCE in patient T cells following SERCA inhibition with thapsigargin, overall indicating basal ER Ca^2+^ store depletion, leading to higher baseline levels of cytosolic (Ca^2+^) in patient T cells compared with paired HD (Fig. 4). This was not associated with constitutively active SOCE (Fig. S1, H and I). Our findings are largely consistent with a recent study looking at the mechanism of three neuropathy-causing missense *ITPR3* variants, including R2524C, demonstrating constitutive IP_3_R channel opening resulting in reduced ER Ca^2+^ stores, increased basal cytosolic Ca^2+^, and inappropriate SOCE when ITPR3 variants were overexpressed in TKO HEK cell lines (Terry et al., 2022).

No significant defect in Ca^2+^ influx was observed in patient fibroblasts, potential reasons for this include cell-type specific differences in IP_3_R isoform expression, homolog functional dominance, and compensatory mechanisms. A prior study showed that ITPR2 subunits within IP_3_R heterotetramers dictate the outcome of Ca^2+^ signaling in response to ATP, indicating that isoform functional dominance is further influenced by the agonist used to trigger IP_3_R channel opening (Chandrasekhar et al., 2016). Nevertheless, ITPR3 is fairly ubiquitously expressed and Ca^2+^ signaling defects in other cell types must underlie the multisystem disease observed. Cell type- and context-specific differences in intracellular Ca^2+^ signaling have been described extensively (Clapham, 2007). Whereas our study further emphasizes that SOCE is critical for the development of unconventional T cell populations, including Treg, iNKT cells (Lian et al., 2018; Oh-Hora et al., 2013) and γδ T cells, profound lymphopenia of conventional T cells is a hallmark feature in patients with *ITPR3* variants (Table 2 and Fig. 5), but not in CRAC channelopathies (Badran et al., 2016; Byun et al., 2010; Feske et al., 2006, 2010; Fuchs et al., 2012; Lacruz and Feske, 2015; Lian et al., 2018; McCarl et al., 2009; Picard et al., 2009; Schaballie et al., 2015; Yu et al., 2021). Detailed immunophenotyping of various T helper, B cell, and NK cell subsets only showed subtle differences between patients and HDs, which were not convincingly clinically relevant (data not shown). Our study thus further focused on the consequences of IP_3_R channel dysfunction in T cells. Ca^2+^ signaling is crucial for conventional T cell development (Aifantis et al., 2001; Melichar et al., 2013; Nakayama et al., 1992) and, given SOCE via CRAC channels is the predominant mechanism for increase of cytosolic (Ca^2+^) in T cells, the presence of normal absolute T cell counts in ORAI- and STIM-deficient mice (Beyersdorf et al., 2009; Gwack et al., 2008; McCarl et al., 2010; Oh-Hora et al., 2013; Vaeth et al., 2017) and patients (Vaeth and Feske, 2018) is intriguing. It has long been speculated that other Ca^2+^ channels regulate the development of conventional T cells in the thymus. In our study, the lymphopenia documented in P1–P5, despite less severely impaired SOCE, suggests a role for IP_3_R-mediated, but SOCE-independent, Ca^2+^ signaling. *ITPR*1^−/−^/2^−/−^/3^−/−^ TKO mice have small thymi with reduced counts of DP and mature single positive (SP) CD4^+^ thymocytes (Ouyang et al., 2014). Ex vivo co-culture of TKO fetal liver HSPCs with Notch ligand-expressing stromal cells show a developmental block with the accumulation of DN T cells. Using similar ex vivo T cell differentiation assays, we show that bone marrow-isolated HSPCs from two patients carrying heterozygous missense *ITPR3* variants develop into DP T cells (Fig. 6). This most likely reflects residual IP_3_R function in contrast to the complete LOF in the TKO mice. Likewise, thymus tissue from another patient in this study did not show lymphodepletion on histopathological examination. Together with the near absence of circulating RTEs in patients with *ITPR3* variants, we concluded that IP_3_-mediated Ca^2+^ signaling within developing thymocytes is crucial for efficient T cell differentiation at later maturation stages, which could include the transition from DP to SP thymocytes, positive and negative selection, post-selectional maturation, and/or the egress of naïve T cells into the periphery. When this is restored via HCT in P1–P3 and P5, there is recovery of T cell immunity with good proportions of naïve T cells, confirming that the immune defect is primarily of hematopoietic origin (Fig. 6). However, further work is necessary to determine whether thymic stromal cell development or function is impaired in addition to the T cell intrinsic defect we describe here. As expected, the pathologies outside of the immune system, including the ectodermal and neurological features, did not improve after HCT.

In addition to impaired thymic output, the patients’ lymphopenia was associated with an abnormal phenotype of the remaining peripheral T cells, specifically with increased expression of perforin, granzyme, and markers of exhaustion, though some of these features may, in part, reflect their predominance of effector and memory cells (Fig. 5). Although, when assessed at the protein level we did not detect reduced cytokine production, which has been observed in STIM1, ORAI1, and CRACR2A deficiencies (Feske et al., 2010, 2012; Rice et al., 2021), we did find evidence of subtly impaired nuclear translocation of NFAT and transactivation of NF-κB and NFAT target genes, including *IL2* and *IFNG*, consistent with the role for SOCE in their nuclear translocation and subsequent transcriptional activity (Berry et al., 2018; Dolmetsch et al., 1997; Neumann et al., 2023). We postulated that the degree of SOCE impairment, and consequently the quantity and longevity of nuclear NF-κB and NFAT translocation (Feske et al., 2000), might underlie the differences in cytokine production observed between these Ca^2+^ signaling disorders, and this will be addressed as part of future studies. Impaired functional responses to T cell activation, were demonstrated by a defective ability to proliferate (Fig. 7), which is a common feature of the known Ca^2+^ signaling defects underlying IEIs. As has been demonstrated in the context of STIM1/2 deficiency and blockade of downstream signaling via calcineurin inhibition, we found evidence of an abnormal metabolic signature in T cells with variant ITPR3 proteins (Fig. 7), which probably underlies their inability to proliferate normally in response to TCR stimulation. Abolished SOCE in the context of STIM1/2 deficiency and calcineurin inhibition have both been shown to reduce the expression of several metabolite transporters and enzymes, leading to impaired glycolysis and OXPHOS (Vaeth et al., 2017). In the context of IP_3_R channel dysfunction, we similarly found aberrant expression of metabolic enzymes involved in glycolysis and OXPHOS, particularly in CD8^+^ T cells, in keeping with the more severe phenotype in CD8^+^ versus CD4^+^ T cells.

We also noted several distinct findings, which may either be primary defects explained by SOCE-independent functions of IP_3_R-mediated signaling or secondary defects in the context of the exhausted T cell phenotype. For example, we found evidence of impaired upregulation of fatty acid metabolism via GSEA and protein expression of ACC1, an enzyme involved in fatty acid biosynthesis that is particularly important for CD8^+^ T cell homeostasis (Lee et al., 2014). We also found reduced expression of ATP5A, which was notably normal in STIM1/2 deficiency and after calcineurin inhibition (Vaeth et al., 2017). ATP5A is a subunit of mitochondrial ATP synthase Complex V whose expression is normally increased during T cell activation to support OXPHOS (Ahl et al., 2020) and whose function is dependent on Ca^2+^ via ER-mitochondrial membrane contact sites (Phillips et al., 2012). We hypothesize that reduced ATP5A expression may reflect impaired mitochondrial biogenesis in response to TCR stimulation, a process known to depend on mitochondrial Ca^2+^ uptake via the mitochondrial Ca^2+^ uniporter (Liu et al., 2020). Consistent with this, GSEA showed downregulation of multiple mitochondrial pathways including mitochondrial biogenesis, TCA cycle enzymes, and mitochondrial genes. Finally, HK1, which we found to be decreased in both resting and activated patient CD8^+^ T cells, catalyzes the conversion of glucose to glucose-6-phosphate in the cytosol and further promotes OXPHOS by associating with VDAC at sites of ER–mitochondrial contact. Interestingly, HK1 has also been shown to be of particular importance for CD8^+^ T cells in the context of recall responses (Bantug et al., 2018). A detailed study of the impact of these *ITPR3* variants on T cell metabolism will be the focus of future work.

Through in-depth clinical, immunological, and molecular characterization of these five unrelated patients with heterozygous missense *ITPR3* variants, we described a novel syndromic IEI due to abnormal Ca^2+^ dynamics in T cells. We documented impaired SOCE underlying the clinical and immunological features that overlap with CRAC channelopathies. Extensive in silico analysis of *ITPR3* variants, together with in vitro modeling using gene-edited T cell lines, indicate that the CID-causing variants actively disrupt IP_3_R channel function resulting in leakiness with ER Ca^2+^ store depletion. We further provide unique insights into the regulation of Ca^2+^ signaling in T cells by dissecting SOCE-dependent and SOCE-independent defects in T cell development, function, and metabolism.

## Materials and methods

### Case reports

Ethical research approval was obtained from the London Bloomsbury Research Ethics Committee (REC 06/Q0508/16). The 100,000 Genomes Project by GEL (Caulfield et al., 2017) is covered by REC 14/EE/1112. Participants gave informed consent to participate in the study, and clinical data was anonymized.

P1 was born to non-consanguineous parents and presented at 7 years of age with severe warts. She later developed impetigo and treatment refractory molluscum contagiosum. She was found to be profoundly T cell lymphopenic with absent naïve T cells and TRECs, restricted TCRVβ repertoire, and absent proliferation in response to PHA, but was only mildly B cell lymphopenic with normal humoral responses (Table 2). She was persistently thrombocytopenic, with a paucicellular but otherwise normal marrow. Initial genetic testing using a next-generation sequencing panel containing known IEI genes did not reveal any variants and she was diagnosed with genetically undefined CID. A thymic stromal cell defect was considered, but further investigation revealed a normal-sized thymus with unremarkable histopathology. At 9 years, she underwent HCT without complications from a matched sibling donor (MSD) after reduced intensity conditioning (RIC). She achieved stable mixed chimerism with normal T cell counts and good proportions of naïve T cells, and significant TREC levels (Fig. 6 D and Table 2). Immunoglobulin replacement therapy (IgRT) has not been discontinued. After HCT, her warts and molluscum resolved completely. She is now 24 years old and remains well without further complications.

P2 was born to non-consanguineous parents and presented in infancy with feeding difficulties and repeated lower respiratory tract infections (LRTI). She had profound T cell lymphopenia, low naïve T cells, few RTEs, very low TRECs, a restricted TCRVβ repertoire, and absent proliferation in response to PHA (Table 2). She had mild hypogammaglobulinaemia with preserved vaccination responses against tetanus. She initially remained well on conservative management with antimicrobial prophylaxis and IgRT and was able to clear adenovirus gastroenteritis and viral upper RTIs (URTIs). Initial genetic investigations, including singleton WES, were inconclusive and she was diagnosed with genetically undefined CID with features of ED including microdontia and peg-shaped lower incisors (Fig. 1 A). At the age of 3 years, she was admitted with a prolonged RSV-positive URTI. As her CD3^+^ T cell counts were persistently below 300 cells/μl and in light of the newly diagnosed ITPR3 defect in the context of this study, she underwent an RIC HCT from an MSD 2 years ago and is showing successful immune reconstitution (Fig. 6 D and Table 2). P2 additionally suffers from several significant non-immunological clinical issues including aspiration of thin fluids regurgitation and chronic constipation. Because of persistent tiptoeing, she underwent neurological investigation at the age of 3 years, which was suggestive of a CMT-like chronic motor neuronopathy affecting the limbs, without evidence of demyelination.

P3 was born to non-consanguineous parents. He suffered from eczema, asthma, frequent mild RTIs, and recurrent ear infections during infancy and childhood. At 11 years old, he was admitted with pneumonia. A year later, he had recurrent pneumonia, splenomegaly, lymphadenopathy, and low-level EBV viraemia. In parallel, he was found to be panlymphopenic (Table 2) with reduced naïve T cells, low TRECs, and abnormal TCRVβ spectratyping. Proliferation in response to PHA was impaired. IgG and IgM levels were mildly low, but humoral responses to vaccines were preserved (Table 2). There were no coding variants in a panel of known IEI genes and he was initially diagnosed with undefined CID with lymphoproliferation and ED, including hypo- and microdontia. He repeatedly presented with transient lymphadenopathy without evidence of lymphocyte clonal expansions on multiple biopsies. He became progressively hypogammaglobulinaemic and was started on IgRT. At the age of 22 years, he represented with B symptoms, persistent cough, hepatosplenomegaly, and widespread lymphadenopathy. He was diagnosed with stage 4B T cell rich, B cell lymphoma with bone marrow, and extensive extranodal involvement. He had concurrent nodular lymphocyte predominant Hodgkin lymphoma (NLPHL) on lymph node biopsies and an EBV-positive mucocutaneous ulcer of the tongue. He was treated to complete remission and then proceeded to an RIC HCT from a matched unrelated donor. He achieved stable mixed chimerism and slowly recovered normal T cell counts with normal proportions of naïve T cells (Fig. 6 D and Table 2) and clinical improvement. EBV was undetectable in blood.

P4 and her fraternal twin were born at 31 wk of gestational age to non-consanguineous parents. She required non-invasive respiratory support for her first month of life and subsequently suffered from wheezing, paroxysmal coughing, and recurrent LRTIs. Although these symptoms improved over time, she was found to be bronchiectatic. She also has ED features with small conical teeth, thin hair, sparse eyebrows, frail nails, and hypohidrosis; NF-κB-mediated cytokine production in response to TLR agonists was normal. She was found to be T cell lymphopenic (Table 2), was diagnosed with undefined CID, and antibiotic prophylaxis was started. She is now 18 years old with persistent T cell lymphopenia and worsening proliferative response to PHA (Fig. 7 B). She has recurrent warts but overall is clinically well under conservative management (Table 1). Protective antibody titers were documented after tetanus, haemophilus, and pneumococcal vaccinations, but with the rapid decay of levels.

P5 was born after in vitro fertilization with donor sperm. She first presented at 14 mo of age with a rash and mild rectal bleeding. She was found to be thrombocytopenic and was diagnosed with ITP, which resolved spontaneously. At three and a half year years old, she was referred for investigation of failure to thrive, mild developmental delay of gross motor skills, hypermobility, microcephaly, syndromic features including ED with thin hair, slightly conical teeth, and hypohidrosis (Fig. 1 A). She had a significant history of recurrent LRTIs due to *Streptococcus pneumoniae* and *Haemophilus influenzae*. She was found to have profound T cell lymphopenia and mild B cell lymphopenia (Table 2), was diagnosed with CID, and commenced antibiotic prophylaxis. Cellular responses to TLR agonists were not impaired. She remained lymphopenic with progressively declining B cell counts. After vaccination, antibody titers against tetanus and pneumococcal vaccine serotypes were documented within the lower normal ranges. Whilst largely remaining free from infection under initial conservative management, imaging showed evidence of early bronchiectasis. She was also diagnosed with chronic demyelinating motor sensory neuropathy with progressively worsening gait disturbance, tiptoeing, and distal muscle weakness. In light of the overall clinical picture, a Ca^2+^ channelopathy, such as STIM1 or ORAI1 deficiency, was suspected, but not genetically confirmed after initial WGS analysis. At 12 years old, she was diagnosed with EBV-related stage 4 B cell lymphoma with central nervous system involvement. Upon achieving remission after chemotherapy, she received a RIC HCT from a matched unrelated donor 6 mo ago for CID due to ITPR3 deficiency diagnosed in the context of this study and is showing early signs of successful immune reconstitution.

Informed consent for participation in this study was obtained in accordance with local research ethics and the Declaration of Helsinki. The experiments included in this manuscript were performed in institutions in the United Kingdom in conformity with local regulations.

### Sequencing

For P1 and her family, whole-exome capture was performed using Agilent capture baits (SureSelect_Human_All_Exon_50 Mb), and samples were indexed and sequenced on the Illumina Sequencing platform at the Wellcome Sanger Institute generating on average 5.87 Gb of raw sequence/sample. Data were aligned to the 1,000 genomes reference (hs37d5) with BWA-mem (v0.7.17-r1188) resulting in an average of 98× coverage. Variants were called using SAM-tools and bcftools (v1.15). Since the proband was the only affected individual within the pedigree, we filtered her germline variant calls to remove variants found in her parents and siblings. Analysis of variants in this way identified a single missense *ITPR3* variant of high quality that appeared to be a de novo variant. The sequencing data for this kindred has been deposited on the European Genome-phenome Archive (EGA study accession number: EGAS00001000099); https://ega-archive.org/datasets/EGAD00001005263.

For P2 and her unaffected parents, WGS sequencing at a minimum 30X mean coverage was conducted by Centogene. They did not report any pathogenic or likely pathogenic variants in considered candidate genes. The sequencing data was subsequently released for re-analysis. Reads were aligned to GRCh38 using bwa v0.7.17 (Li and Durbin, 2009) and duplicated reads were marked using samblaster v0.1.26 (Faust and Hall, 2014). Small variants were identified from single individual BAM files using deepvariant v1.0.0 (Poplin et al., 2018) and single individual gVCFs were merged in a trio VCF using GLnexus v1.2.7 with deepvariantWGS optimized settings (Yun et al., 2021). Variants were filtered retaining only variants with quality above 20 and at least one individual with GQ ≥10 and then normalized using bcftools norm v.1.10.2. We used SnpEFF v4.3 (Cingolani et al., 2012) and vcfanno (Pedersen et al., 2016) to annotate the filtered VCF with gene level consequences, allele frequency from general population, allele frequency from a cohort of 300 WGS samples processed with the same pipeline, low complexity regions (Li, 2014), and impact predictions scores. High confident de novo variants (DNM) were determined according to the following criteria: DP ≥10 in all individuals, not located in a low-complexity region, GQ ≥20 in both parents, GQ ≥30 in the proband, alternate allele fraction in the proband between 0.2 and 0.8 and no alternate alleles observed in the parents. Among these, we identified as candidate DNM variants the protein-changing variants that were rare (AF < 0.01) in the 1,000 G/gnomAD (v3.1 or v4.1) populations and with AF <0.1 in the internal control cohort. A ranked list of genes potentially relevant to the family phenotype was calculated based on the HPO profile using GADO (Deelen et al., 2019).

P3–P5 were identified from the GEL Rare Diseases database (v11), which includes genomes from 86,978 participants, where 71,369 were enrolled in the Rare Diseases program (Caulfield et al., 2017). This includes probands with genetically undefined disorders and affected/unaffected relatives, distributed across 34,948 pedigrees of which ∼35% (12,219) are trios and a further ∼17% are duos or larger pedigrees that include one parent. Data are available for approved researchers via the National Genomic Research Library (NGRL); https://www.genomicsengland.co.uk/research/members.

The *ITPR3* variants identified were validated by Sanger sequencing using genomic DNA, which was PCR amplified and cleaned up using Taq DNA polymerase (Sigma-Aldrich) and the DNA Clean-up and Concentrator-5 Kit (Zymo Research), respectively. Sequencing was performed on an ABI-3730 DNA Analyser using BigDye Terminator v3.1 chemistry (Applied Biosystems). The following primers were used ITPR3_gDNA_P1_F: 5′-CAA​CGG​GGA​CAA​CGT​GAG​G-3′; ITPR3_gDNA_P1_R: 5′-′GGA​AGG​GTG​ACA​AGA​CCC​GT-3′; ITPR3_gDNA_P2-5_F: 5′-AGT​CCA​CGC​TTT​CCT​AAC​CCA-3′; and ITPR3_gDNA_P2-5_R: 5′-TCA​GAT​GAC​GTT​CCA​GCC​CC-3′. Returned sequences were visualized using SnapGene Viewer (v5.3.2).

### Cell culture and stimulations

PBMCs were isolated from blood by density gradient centrifugation using Ficoll Paque Plus (Sigma-Aldrich). Cell suspensions enriched for T cells were achieved through magnetic bead negative selection with MACS MicroBeads (Miltenyi) targeting CD19^+^ cells and CD16^+^ cells from whole blood using the autoMACS Pro Separator. Lymphoblastoid cell lines (LCLs) were generated from peripheral blood by EBV transformation using standard methods. PBMCs, T cells, and LCLs were cultured at a density of 10^6^ cells/ml in RPMI-1640 GlutaMAX culture medium (Gibco) supplemented with 10% Fetal Bovine Serum (FBS) (Gibco) and 1% penicillin/streptomycin (P/S) (Gibco). When frozen PBMCs were used, they were resuspended in RPMI + 10% FBS + 1% P/S after thawing and kept overnight at 37°C, 5% CO_2_, and 95% humidity before use. Dermal primary fibroblasts were grown from skin biopsies and then maintained in DMEM GlutaMAX (Gibco) supplemented with 10% FBS and 1% P/S. Stimulations were performed, at the doses stated and for the indicated times, with thapsigargin (Sigma-Aldrich or Invitrogen), anti-CD3 (BD), AffiniPure F(ab’)2 Fragment Goat Anti-Mouse IgG (Jackson ImmunoResearch), ionomycin (Sigma-Aldrich), phorbol 12-myristate 13-acetate (PMA) (Sigma-Aldrich), CD3/CD28 Dynabeads (Gibco), Ultra-LEAF Purified CD3 and CD28 (Biolegend), OKT3 (EBioscience), PHA (Bio stat), IL-2 (Pharmacy), and anti-FAS IgM (Millipore).

### DNA and RNA extraction, cDNA synthesis, real-time PCR (RT-qPCR), and cDNA Sanger sequencing

Genomic DNA (gDNA) was isolated from whole blood, primary T cells, and primary fibroblasts by phenol/chloroform extraction. RNA extraction from primary T cells and fibroblasts was performed using either the RNeasy Micro or QIAamp RNA Blood Mini Kits (Qiagen). gDNA was removed using DNAseI treatment. Complementary DNA (cDNA) was synthesized using the SensiFAST cDNA Synthesis Kit (Bioline). RT-qPCR was performed using the SensiFAST SYBR Hi-Rox Kit (Bioline) and a StepOnePlus real-time thermal cycler (Applied Biosystems). For *ITPR1*, *ITPR2*, and *ITPR3*, previously published primer sequences were used (Sankar et al., 2014), and for *ACTB,* the following primers were used, ACTBF: 5′-AGA​GCT​ACG​AGC​TGC​CTG​AC-3′; ACTBR: 5′-AGC​ACT​GTG​TTG​GCG​TAC​AG-3′. The delta CT method was used to determine relative expression. To assess whether the variant *ITPR3* alleles were specifically expressed, cDNA was end-point PCR amplified, cleaned up, and Sanger sequenced as described above using the following primers: ITPR3_cDNA_P1_F: 5′-GTA​TGG​CAG​TGT​GAT​CCA​GCT​C-3′; ITPR3_cDNA_P1_R: 5′-TGA​AGC​GGT​ACA​AGC​CAT​TCC-3′; ITPR3_cDNA_P2-5_F: 5′-GGG​ACA​AGA​TGG​ACT​GTG​TCT​C-3′; ITPR3_cDNA_P2-5_R: 5′-CCA​GTC​CAG​GTT​CTT​GTT​CTT​GA-3′.

### Protein extraction and western blotting

Total protein was extracted from cells with RIPA buffer (Sigma-Aldrich) supplemented with Complete Mini Protease Inhibitor Cocktail (Roche). Protein fractions were separated by SDS-PAGE and electrotransferred on Millipore Immobilon-P membranes (Merck). The following primary antibodies were used: mouse anti-ITPR3 (BD Biosciences) and mouse anti-GAPDH (Santa Cruz Biotechnology) IgG antibodies. Antibody binding was detected by incubation with HRP-conjugated anti-mouse IgG antibody (GE Healthcare Life Sciences), with the ECL system (Thermo Fisher Scientific ). Quantification of band intensity was determined using ImageJ.

### Generation of IP_3_R3 variant cell lines

*ITPR3* KO was achieved in HEK293T and Jurkat cell lines using High Fidelity Cas9 protein complexed to three separate guide ribonucleic acid (gRNA) (5′-GCC​UGC​AGG​GAG​ACG​UGG​UG, CCU​UGU​ACU​CGU​CAC​ACG​UC-3′ and 5′-CUC​CCA​GAG​AGC​AUU​GGA​GC-3′). The design and synthesis were carried out by Synthego (Gene Knockout Kit v2). Both target cell lines were expanded for several days in their respective culture media, and subsequently, one million cells were washed and resuspended in Maxcyte Electroporation Buffer (Cytiva). Concurrently, Cas9 and gRNA were incubated together at 37°C for 15 min. They were then added to the cell solution, achieving concentrations of 2 and 4 μM, respectively. The cell solution was placed into an OC-25 cuvette (Maxcyte) and the Maxcyte GT platform was used to electroporate the cells, as described elsewhere (Bahal et al., 2024). Five days after electroporation, DNA was extracted from cell aliquots and the area around the cut sites was PCR amplified with forward primer 5′-TCT​CTA​GGG​GGC​TCA​GCT​TG-3′ and reverse primer 5′-TGG​ACC​CTC​TCT​GGC​CTT​TT-3′. The amplicon was sequenced with primer 5′-TGG​ACC​CTC​TCT​GGC​CTT​TT-3′ and analyzed via the ICE Analyses online tool (Synthego; https://ice.synthego.com/#/) to determine CRISPR/Cas9 cut efficiency. Single cells were isolated by fluorescence-activated cell sorting (FACS) on the BD FACSAria Cell Sorter, and individual clones were cultured. *ITPR3* KO was further confirmed by qPCR and western blotting, as described above, in selected HEK293T and Jurkat clones.

The pEF-DEST51-modi vector containing human WT *ITPR3* cDNA was a gift from Prof D. Adams (Wellcome Sanger Institute, Cambridge, UK), and the WT *ITPR3* cDNA was subcloned into a lentiviral vector which was available in our laboratory. The patient *ITPR3* variants were introduced in both vectors by site-directed mutagenesis using the Q5 Site-Directed Mutagenesis Kit protocol (NEB) using primers designed by the NEB online design software, NEBaseChanger. For all vectors, vector size was assessed by gel electrophoresis, and *ITPR3* and adjacent DNA were verified by Sanger sequencing. WT and variant lentiviral vector supernatants were produced as described elsewhere (Schejtman et al., 2020).

ITPR3 KO HEK293T cells were transiently transfected with the pEF-DEST51-modi vectors containing WT or variant *ITPR3* for 72 h following the PolyFect Transfection Reagent protocol by Qiagen. ITPR3 KO Jurkat cells were infected with WT and variant lentiviral vector supernatants for 24 h with a multiplicity of infection (MOI) of 20. Single cells were isolated 48 h later by FACS on the BD FACSAria Cell Sorter and clones were expanded for 3 wk. The frequency of integrated *ITPR3* copies was assessed by digital droplet PCR in individual clones as described elsewhere (Izotova et al., 2021). Clones with *ITPR3* integration were expanded, and stable ITPR3 expression was inferred by confirming persistent *ITPR3* transcription by qPCR, repeatedly performed alongside functional testing.

### In silico modeling of ITPR3 variants

The variants were modeled in silico using published cryo-EM solved structures for ITPR3 and PyRosetta (Chaudhury et al., 2010; Paknejad and Hite, 2018). Each of the structures was energy-minimized using the LocalRelax mover restrained to their respective electron density maps and with a correctly protonated topology for the IP_3_ ligand (Wang et al., 2016). An active form of ITPR3 was constructed by threading using RosettaCM and ITPR1 PDB:6MU1 as a template (Fan et al., 2018; Song et al., 2013). Unless specified, the variants were introduced to the first chain only and energy-minimized again in the 12-Å radius neighborhood using the ref2015 scorefunction. Variants from the gnomAD database were from the version 3.1 control dataset (Karczewski et al., 2020). The interactive figure (available at https://michelanglo.sgc.ox.ac.uk/r/itpr3) was produced in Michelaɴɢʟo (Ferla et al., 2020). For the analysis of the transcriptomic distance between variants, we converted genomic coordinates to exonic coordinates and then calculated the distance of each variant to the closest other *ITPR3* variant in the gnomAD database. The significance of the difference between variants identified in P1–P5 and control gnomAD variants was assessed using the Wilcoxon rank sum test.

### Multiple sequence alignments (MSA)

For the MSA, the following protein sequences from UniProt were used: Q14643, Q14573, A0A0R4IH08 (Trembl entry, but the Swissprot F1R1L5 is a fragment), F8W4B1, E7FB06, A0A4W3JHJ0, A0A4W3JQ24, A0A4W3GHU7, and P29993. Additionally, three NCBI sequences were used: XP_032819189.1 (absent in UniProt), XP_022786205.1 (full-length unlike Trembl A0A2B4T0S0), and XP_021323455.1 (full-length unlike Trembl F1QW18), representing the genes from *Homo sapiens*, *Danio rerio*, *Callorhinchus milii*, *Petromyzon marinus*, *Drosophila melanogaster,* and *Stylophora pistillata.* MSA plot was generated with Plotly.

### Flow cytometry analysis and FACS

Cell surface antigens were labeled using fluorophore-conjugated antibodies for 30 min at 4°C or room temperature (RT) depending on the assay. For experiments requiring the analysis of intracellular antigens, fixation and permeabilization were achieved using the ADG Fix/Perm (An der Grub), Human FoxP3 Buffer Set (BD), or the Cytofix/Cytoperm (BD) kit, according to manufacturers’ instructions. Cell viability was assessed using either 7AAD, Zombie UV, or Red Fixable Viability Kits (BioLegend), as per manufacturer’s instructions. Cells were acquired ± sorted using either BD Biosciences’ FACS Aria III, FACSCalibur, or LSR II flow cytometers or Miltenyi’s MACSQuant flow cytometer. Data were analyzed and plots were produced using FlowJo v10.7.2 or Infinicyt v2 (Cytognos). UMAP plots were generated using the UMAP plugin in FlowJo. Assay-specific adaptations to this basic protocol and particulars of analysis are detailed in the relevant methods sections below. A list of antibodies used for flow cytometry experiments is provided in Table S2. Key gating strategies are shown in Fig. S2.

### Ca^2+^ flux in T cells

To assess cytosolic Ca^2+^ levels at baseline and in response to various stimuli, the fluorescent Ca^2+^ indicators Indo-1 and Fluo-4 AM were used in flow cytometric assays. Indo-1 is a ratiometric Ca^2+^ indicator, which changes its emission wavelength when bound by Ca^2+^ from 485 to 405 nm, allowing for analysis of bound/unbound Indo-1 and thus cytosolic (Ca^2+^) dynamics. Fluo-4 AM is a non-ratiometric Ca^2+^ indicator that emits a green fluorescent signal (506 nm) upon binding cytosolic Ca^2+^.

For assays using Indo-1, PBMCs, patient-derived CTLs, or gene-edited Jurkat T cell lines were resuspended in phosphate-buffered saline (PBS) at 10^7^ cells/ml and labeled with 3 μM Indo-1 for 30 min at 37°C. Cell surface staining was performed at RT for CD45, CD8, and CD4. Thereafter, 10^6^ PBMCs per condition were washed and resuspended in 500 μl of PBS without Ca^2+^ (Gibco) or with 0.9 mM CaCl_2_ (Sigma-Aldrich). For TCR stimulation, cells were preincubated with 0.6 μg/ml anti-CD3 (BD, clone UCTH1) for 10 min at 37°C before acquisition. For all the conditions and stimuli, the samples were acquired for 30 s to establish the baseline ratio of bound/unbound Indo-1. Then, the corresponding stimulus was added: (1) 8.5 μl of F(ab’)2 in the cells preactivated with anti-CD3, or (2) 0.5 μM of thapsigargin. In the Ca^2+^-free media conditions, CaCl_2_ was added after another 60 s at a final concentration of 2 mM. All samples were acquired for a total of 5 min. Ionomycin (Sigma-Aldrich) was added at a concentration of 1 μg/ml. Kinetic plots were generated using the FlowJo kinetics tool gated on live T cells and the mean fluorescence intensity (MFI) with Gaussian smoothing of Indo-1 (Bound/Unbound) ratio was analyzed. For comparison, the Indo-1 ratio obtained at the peak signal was normalized using the baseline values (ratio peak/baseline = mean of the Indo-1 ratio values in the peak/the mean obtained for the baseline). To assess the statistical significance (P < 0.05) of differences observed between patients and HDs, the Mann–Whitney U test was used (SPSS software, version 23; IBM).

For assays using Fluo-4 AM, CTLs were loaded with Fluo-4 AM (Invitrogen) at 1 μg/ml and LIVE/DEAD Fixable Yellow dye (Invitrogen) for 20 min at 37°C, then washed and resuspended in Ca^2+^-free assay medium: Ca^2+^-free HBSS (Gibco) and 5% BSA. Cells were initially acquired for 2 min to establish their baseline Fluo-4 fluorescence. Ionomycin was then added to a final concentration of 2 μg/ml, and after 30 s, cells were acquired again for 2 min to record the peak in Fluo-4 fluorescence. Kinetic plots were generated using the FlowJo kinetics tool gated on live cells, and the MFI of Fluo-4 at baseline and the peak following ionomycin was compared to obtain a ratio of peak/baseline. Values obtained for patient-derived CTLs were normalized to their respective healthy controls and expressed as fold-change in Fluo-4 AM ratio in patient CTLs over HDs, and Welch’s *t* test was used to assess for statistical significance.

### Ca^2+^ imaging in fibroblasts

72 h prior to Ca^2+^ imaging, fibroblasts were seeded onto fluorodishes at 1 × 10^4^ cells per dish. For imaging of cytoplasmic Ca^2+^, cells were washed once with DMEM containing HEPES (Gibco), then incubated with 1 μM Indo-1 AM (Invitrogen) and 0.02% Pluronic F-127 (Sigma-Aldrich) for 30 min at 37°C. Fibroblasts were then washed and incubated for a further 20 min in DMEM at 37°C to allow de-esterification of intracellular AM esters. Cells were imaged using a UV-vis Zeiss LSM 880 confocal equipped with a 20× objective. Indo-1 fluorescence was excited at 355 nm and emission was measured simultaneously between 371 and 447 nm for Ca^2+^-bound Indo-1 and between 468 and 567 nm unbound Indo-1. Time-lapse images were acquired at 2 Hz. To investigate IP_3_-mediated signaling, fibroblasts were stimulated using 100 μM ATP. Following the depletion of ER stores, CaCl_2_ (Sigma-Aldrich) was added to cells at a final concentration of 1.2 mM. Images were analyzed using ImageJ/FIJI. Regions of interest (ROI) were manually selected for each cell, and MFI was quantified for all ROIs in each channel. The background was subtracted and ratios between the emission signals of bound/unbound Indo-1 were calculated over time. The resulting ratioed traces representing cytosolic (Ca^2+^)_c_ levels have been plotted. GraphPad Prism was used to carry out the area under the curve (AUC) analysis, and peak amplitude and area have been plotted.

### Immunophenotyping

The T cell phenotype was analyzed in peripheral blood of P2, P3, P4, and P5, as well as in 10 HDs: five adults (25–45 years) and five pediatric HDs (3, 5, 6, 13, and 15 years). The staining was performed using previously published protocols (Blanco et al., 2018; Botafogo et al., 2020; Kalina et al., 2012), and the antibodies used are listed in Table S2. Absolute numbers were calculated using BD TruCount tubes (BD). To establish if the patients had alterations in the subsets analyzed, they were compared with the age-matched HDs (adult or pediatric HDs) analyzed in parallel and, when available, with previously published reference data. To assess the statistical significance (P < 0.05) of differences observed between patients and HDs, Mann–Whitney U test was used (SPSS software, version 23; IBM).

### TCRVβ spectratyping

T cell clonality was assessed using TCRVβ chain spectratyping on isolated CD3^+^ cells as previously described (Amrolia et al., 2006) To quantify results, the median number of peaks per Vβ family and the total number of Gaussian Vβ families were counted. According to in-house unpublished data, the spectratype was defined as skewed and abnormal when the number of peaks per Vβ family was <7 and the number of Gaussian families was <10. If present, oligoclonal expansions were also recorded for each Vβ family.

### Apoptosis assay

PBMCs from patients and HDs were cultured at 1 × 10^6^/ml in 96-well plates and incubated at 37°C, 5% CO_2_ for 6–7 days with OKT3 (1 μg/ml) and IL-2 (100 U/ml). The cells were then stimulated for 4 h with either PHA (15 μg/ml) or anti-FAS IgM (5 μg/ml) to trigger apoptosis. Cell surface and DNA staining were performed prior to FACS acquisition to identify cells undergoing apoptosis (AnnexinV^+^7AAD^−^). The fold change in % of Annexin V single positive cells in stimulated versus unstimulated samples was determined. Additionally, baseline apoptosis was determined on day 0 in unstimulated PBMCs.

### Ex vivo T cell differentiation

CD34^+^ HSCs are isolated from patient bone marrow and control cord blood through FACS or immunomagnetic separation and cultured with the OP9/DL1 or MS5/DL1 stromal murine cell line, respectively in a two-dimensional assay (Schmitt and Zúñiga-Pflücker, 2002; Six et al., 2011) or a three-dimensional ATO assay (Seet et al., 2017). Stages of T cell differentiation were assessed weekly for up to 6 wk by immunophenotyping using CD3, CD4, CD8, TCRαβ, and TCRγδ as cell surface markers within 7AAD^−^CD45^+^ cells (Seet et al., 2017; Six et al., 2011).

### CD25 and CD40L upregulation in PBMCs

Whole blood was incubated overnight with PHA (6 μg/ml) and PMA (20 ng/ml). Cell surface staining was performed on day 2 (see Table S2) before FACS acquisition and analysis. Patient samples were always run alongside a HD.

### NFAT nuclear translocation assay

P815 target cells (ATCC, TIB-64) expressing mtagRFP fused with a membrane-targeting domain were seeded onto multiwell slides and cultured overnight in DMEM (Gibco) with 10% FBS at 5 × 10^5^ cells/ml. The next day, the adhered P185 target cells were incubated for 1 h in media with or without 1 μg/ml anti-human CD3ε (clone OKT3; Absolute Antibodies), after which the media was replaced with a serum-free RPMI suspension of 1 × 10^6^/ml CTLs from HD or patients. Conjugation was allowed to proceed for 20 min at 37°C, 10% CO_2_ in the dark. Cells were then fixed in 4% paraformaldehyde at room temperature for 20 min and subsequently permeabilized in 0.1% Triton X-100 in PBS for 8 min and incubated with a blocking buffer (2% BSA in PBS) for 45 min. Primary antibody staining was done overnight at 4°C with rabbit anti-NFAT1 (clone D43B1, diluted 1:200; Cell Signaling Technology) and mouse anti-CD8a (clone 37006, 1:100; R&D Systems). Slide wells were thoroughly washed with blocking buffer before staining with DAPI (5 ng/ml) and secondary antibodies, goat anti-mouse (Ax488, 1:400; Invitrogen), and goat anti-rabbit (Ax647, 1:400; Invitrogen) for 1 h at room temperature. After washing with PBS, coverslips were mounted using SlowFade Glass mountant (Invitrogen) and sealed with nail polish. Slides were imaged on the same day on a spinning disc confocal system (Revolution, Andor) fitted with a CSU-X1 spinning disk unit (Yokogawa) via a DMi8 microscope (Leica). Samples were excited with lasers of 405, 488, 543, and 633 nm wavelength. Images were captured using an iXon Ultra 888 camera and Fusion software (Andor) through a 100× objective lens (NA 1.40; Leica) with z-slices taken every 0.15 μm. 70 × 70 μm fields of view were acquired in a 5 × 5 montage for each sample and maximum intensity projections were generated from images. Single CTL-P185 conjugates were manually cropped and analyzed for MFI of NFAT in the nucleus and in the cytoplasm using a custom ImageJ macro.

### Cytokine production

Thawed PBMCs were stimulated with 1 μg/ml ionomycin and 25 ng/ml PMA (4 h) or CD3/CD28 Dynabeads at a ratio of 1:1 (16 h) in the presence of GolgiPlug (containing Brefeldin A; BD) at 1 μl/ml. An unstimulated sample aliquot (containing GolgiPlug in the absence of any stimuli) was processed in parallel. After stimulation, cell surface and cytoplasmic antigens were labeled using antibodies provided in Table S2. Following FACS acquisition the percentage of positive cells over unstimulated was calculated for each cytokine, after exclusion of naïve T cells.

### Degranulation assay

PBMCs from three non-transplanted patients (P2, P4, and P5) and six HDs were thawed and rested, then stimulated for 6 h with Ultra-LEAF Purified CD3 (2 ng/ml) and CD28 (4 ng/ml) in the presence of CD107a (5 μl/ml) for 6 h at 37°C, 5% CO_2_, and 95% humidity, with 0.7 µl/ml GolgiStop (containing Monensin; BD) for the last 5 h. For each sample, an unstimulated aliquot was processed in parallel. After stimulation, cells were prepared for flow cytometric analysis using the antibodies provided in Table S2. The percentage of CD107a positive cells over unstimulated was calculated for the stimulated samples.

### Proliferation assay

Cells were cultured in 96-well plates at a concentration of 1 × 10^6^/ml (either whole blood or lymphocytes separated from whole blood using lymphoprep), with or without stimulation using PHA (8 μg/ml), OKT3 (1.5 μg/ml), or PMA/ionomycin (respectively, 25 ng/ml and 1 μg/ml). Plates were incubated at 37°C with 5% CO_2_ for 4 days before pulsing for 4 h with tritiated (^3^H) thymidine. The incorporation of tritiated thymidine was measured using a β counter and expressed as counts per minute minus background (unstimulated). Patient samples were always run alongside a HD.

### Analysis of metabolic enzymes

PBMCs (1 × 10^6^cells/ml) were cultured with CD3/CD28 Dynabeads at a ratio of 1:1. Following stimulation for 24 h, cells were prepared for flow cytometric analysis as detailed above. Antibodies used for cell surface staining are included in Table S2. The Cytofix/Cytoperm kit (BD) was used for staining intracellular metabolic enzymes. The purified metabolic antibodies were purchased from Abcam and conjugated with goat anti-rabbit IgG AF647 (Invitrogen). A rabbit IgG isotype control (Invitrogen) was used as the negative control.

### RNA sequencing and analysis

After red blood cell lysis, magnetically sorted T cells (negative fraction, obtained after CD14, CD15, and CD19 positive selection using the Miltenyi AutoMACS Pro Separator) were stimulated with CD3/CD28 Dynabeads at a ratio of 1:1 for 4 or 12 h, or left unstimulated. RNA was extracted as described above and quantified using the RiboGreen RNA assay kit (Invitrogen) and a FLUOstar OPTIMA plate reader (BMG Labtech). The size profile and integrity were analysed on by TapeStation (Agilent, RNA ScreenTape). Input RNA was normalized prior to library preparation and depleted of ribosomal RNA using the NEBNext rRNA Depletion Kit (NEB). Strand-specific libraries were prepared using the NEBNext Ultra II mRNA kit (NEB) and amplified on a Tetrad (Bio-Rad) using in-house unique dual indexing primers (based on [Lamble et al., 2013]). Individual libraries were quantified using Qubit reagents (Invitrogen), size profiled on the TapeStation, normalized, and pooled. The pooled library was diluted to 10 nM for storage. The 10 nM library was denatured and further diluted prior to loading on the sequencer. Paired-end sequencing was performed using a Novaseq 6000 platform at 150 base pair paired-end read configuration. For analysis of RNA-sequencing data, reads were trimmed using Trimmomatic and aligned to the mouse genome (mm10) using STAR (version 2.7.3a) (Dobin et al., 2013). Reads were assigned to genes using HTSeq (Anders et al., 2015). Differential analysis was conducted using edgeR (Robinson et al., 2010). Gene set enrichment analysis was undertaken according to Subramanian et al. (2005) using the following gene sets: NFAT Q4 01, NFKAPPAB 01, Zheng bound by FOXP3, reactome fatty acid metabolism, GOCC TCA cycle enzyme complex, reactome mitochondrial biogenesis, Wong mitochondrial gene module, and GOBP protein import into the mitochondrial matrix. We assessed transcription factor motif enrichment in differentially expressed genes using SCENIC (Aibar et al., 2017). Motif analysis was restricted to 5 kb within the transcription start site of genes and significant motif enrichment was defined using the default threshold of 3 for normalized enrichment scores.

### Online supplemental material

Fig. S1 shows data regarding ITPR expression in fibroblasts and Ca^2+^ flux in patient fibroblasts, primary T cells, gene-edited Jurkat T cells, CTLs, and ex vivo differentiated T cells. Fig. S2 shows gating strategies used in flow cytometric assays. Fig. S3 shows additional functional T cell assays performed using the patient primary T cells. Table S1 shows data relating to T cell subsets in patients alongside reference data generated from pediatric HD. Table S2 details antibodies used in flow cytometry experiments.

## Supplementary Material

Table S1 shows T cell subsets in patients with heterozygous *ITPR3* mutations.

Table S2 shows antibodies used in flow cytometry experiments.

SourceData F2 is the source file for Fig. 2.

SourceData F4 is the source file for Fig. 4.

SourceData FS1 is the source file for Fig. S1.

## Data Availability

Clinical and clinical laboratory data are stored within the firewall of the UK National Health Service. In line with recommendations from the Caldicott Guardian and the Information Governance advisory services from the Directorate of Research and Innovation at Great Ormond Street Hospital for Children NHS Foundation Trust for cohorts of <10 GOSH patients, personally identifiable genetic data (personal data) have not been deposited into public archives. WGS data from P2 trio and RNA sequencing data from P2, P4, and P5 are available from the corresponding author upon reasonable request. All the other data needed to evaluate the conclusions of the manuscript are present in the manuscript or the supplemental data.
